# Multifunctional SiC@SiO_2_ Nanofiber Aerogel with Ultrabroadband Electromagnetic Wave Absorption

**DOI:** 10.1007/s40820-022-00905-6

**Published:** 2022-07-28

**Authors:** Limeng Song, Fan Zhang, Yongqiang Chen, Li Guan, Yanqiu Zhu, Mao Chen, Hailong Wang, Budi Riza Putra, Rui Zhang, Bingbing Fan

**Affiliations:** 1 School of Materials Science and Engineering, Zhengzhou University, Zhengzhou, 450001 Henan People’s Republic of China; 2 School of Materials Science and Engineering, Zhengzhou University of Aeronautics, Zhengzhou, 450015 Henan People’s Republic of China; 3 College of Engineering, Mathematics and Physical Sciences, University of Exeter, Exeter, EX4 4SB UK; 4 Research Center for Metallurgy, National Research and Innovation Agency, South Tangerang, 15315 Banten Indonesia; 5 State Key Laboratory of Crystal Materials, Shandong University, Jinan, 250100 Shandong People’s Republic of China; 6 School of Materials Science and Engineering, Luoyang Institute of Science and Technology, Luoyang, 471023 Henan People’s Republic of China

**Keywords:** Multifunctional, SiC@SiO_2_ nanofiber aerogel, Chemical vapor deposition, Electromagnetic wave absorption, Ceramic materials

## Abstract

Traditional ceramic materials are generally brittle and not flexible with high production costs, which seriously hinders their practical applications. Multifunctional nanofiber ceramic aerogels are highly desirable for applications in extreme environments, however, the integration of multiple functions in their preparation is extremely challenging. To tackle these challenges, we fabricated a multifunctional SiC@SiO_2_ nanofiber aerogel (SiC@SiO_2_ NFA) with a three-dimensional (3D) porous cross-linked structure through a simple chemical vapor deposition method and subsequent heat-treatment process. The as-prepared SiC@SiO_2_ NFA exhibits an ultralow density (~ 11 mg cm^− 3^), ultra-elastic, fatigue-resistant and refractory performance, high temperature thermal stability, thermal insulation properties, and significant strain-dependent piezoresistive sensing behavior. Furthermore, the SiC@SiO_2_ NFA shows a superior electromagnetic wave absorption performance with a minimum refection loss (*RL*_min_) value of − 50.36 dB and a maximum effective absorption bandwidth (*EAB*_max_) of 8.6 GHz. The successful preparation of this multifunctional aerogel material provides a promising prospect for the design and fabrication of the cutting-edge ceramic materials.

**Supplementary Information:**

The online version contains supplementary material available at 10.1007/s40820-022-00905-6.

## Highlights

- A multifunctional SiC@SiO_2_ nanofber aerogel (NFA) was successfully prepared, which exhibits ultra-elastic, fatigue-resistant, high-temperature thermalstability, thermal insulation properties, and signifcant strain-dependent piezoresistive sensing behavior.
- The SiC@SiO_2_ NFA shows excellent electromagnetic wave absorption performance with a minimum refection loss value of −50.36 dB and a maximum effective absorption bandwidth of 8.6 GHz.

**Supplementary Information:**

The online version contains supplementary material available at 10.1007/s40820-022-00905-6.

## Graphical abstract

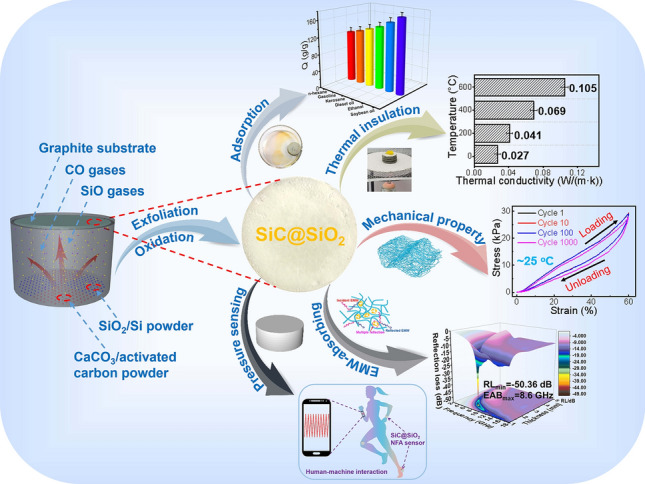

## Introduction

Ultralight ceramic aerogels have the characteristics of low density, high porosity, large specific surface area, excellent thermal and chemical stability, which hold great potentials in the applications of energy storage [1], catalytic [2], thermal insulation [3], environmental [4, 5], electromagnetic wave (EMW) absorbing [6, 7] and electromagnetic interference shielding [8–10] fields. However, conventional ceramic aerogels typically have poor mechanical properties because they are composed of necklace-like linked nanoparticles [11]. Polymer or carbon aerogels can achieve superelasticity, but there are few literature reports on the realization of superelasticity based only on ceramic component aerogels [12, 13]. It is due to the fact that the elastic bending strain of ceramics is not as good as that of polymers or carbons, thereby achieving superelasticity in ceramic aerogels is quite challenging [5]. In addition, most of the currently reported ceramic aerogels still lack structural integrity and stable fiber-to-fiber cross-linking, which can only exhibit limited elastic deformation, resulting in their poor mechanical properties. Current methods to enhance their mechanical properties usually involved the addition of polymer or carbon components [14, 15], which restricts these aerogels from applications in high temperature or harsh environments owing to the limited heat resistance of the polymer/carbon components [16, 17]. Thus, it is an extremely challenging goal to realize the versatility of ceramic aerogels for their practical applications under various harsh conditions.

The properties of aerogels generally depend on the intrinsic properties, density and cellular structure of the solid components [18]. Maintaining the structural integrity of ceramic aerogels under stress is a prerequisite for practical applications, and among various ceramic materials, nanofibrous ceramic materials exhibit high mechanical efficiency [19]. Notably, SiC nanofibers exhibit excellent physical, chemical, electrical, and optical properties [20–22] and are an ideal candidate for the construction of multifunctional ceramic aerogels. Compared with ordinary one-dimensional (1D) SiC nanowires, the chemical and thermal stability of SiC/SiO_2_ nanofibers is higher [23]. Thus, 3D cross-linked aerogels composed of SiC/SiO_2_ nanofibers have wider applications. This superb structure constructed from SiC/SiO_2_ nanofibers with a high aspect ratio has sparked great interests in the design of multifunctional ceramic aerogels.

Various methods have been proposed to synthesize nanofiber ceramic aerogels, including electrospinning [24], and freeze-drying [19, 25]. However, these strategies still have disadvantages such as expensive or toxic reagents, cumbersome processing routes, strict operating conditions, and complex equipment, which are difficult to produce at a large scale [26]. It is worth noting that CVD is a convenient, simple and sustainable method for fabricating nanofiber ceramic aerogels [27]. Although the as-prepared nanofibrous aerogels possess high compressibility and excellent chemical and thermal stability, their practical application is hindered by expensive raw materials and/or the size limitation of the fabrication tools. Additionally, compared with the reported high-performance absorbing materials, such as magnetic materials [28, 29], carbon materials [30, 31], and conducting polymers [32, 33], SiC matrix materials could be used as a high-temperature absorbing material, however, the narrow effective absorbing bandwidth is an intrinsic problem [34, 35].

Herein, we report the realization of a 3D porous cross-linked SiC@SiO_2_ NFA via a simple CVD method and subsequent heat treatment process by using low-cost raw materials. The SiO_2_ nanolayer is introduced to the surface of the SiC nanofiber through the oxidation process, which not only optimizes its impedance matching to improve the microwave absorbing properties, but also enhances its high-temperature thermal stability.

## Experimental Section

### Raw Materials

Activated carbon (C, analytically grade) and calcium carbonate (CaCO_3_, analytically grade) were obtained from Sigma-Aldrich (USA). Silicon nanopowder (Si, analytically grade, 200 mesh) and silica (SiO_2_, analytically grade, 80 mesh) were procured from Aladdin Co., Ltd (USA).

### Preparation of the SiC@SiO_2_ Nanofiber Aerogel

Firstly, a mixture of activated carbon and CaCO_3_ (C/CaCO_3_, molar ratio = 1:1) was selected as the carbon source, which was ball milled for 5 h at 300 rpm. Then, a mixture of SiO_2_ and Si nanoparticles (molar ratio of Si/SiO_2_ = 1:1) as the silicon source was ball-milling pretreated for 5 h at 300 rpm. Thirdly, the C/CaCO_3_ and Si/SiO_2_ mixtures successively transferred into the graphite crucibles were heated in a nitriding furnace (ZSL 1600X, Zhengzhou Kejing Electric Furnace Co., Ltd, China) set at 1500 °C for 5 h under Ar atmosphere to prepare the SiC nanofiber aerogels (SiC NFA). The graphite crucible was taken out of the furnace after it had cooled down. Fourthly, to exfoliate the SiC NFA, a graphite lid deposited with the nanofiber aerogel was calcined at 700 °C for 2 h in air. Finally, the resulting aerogel was calcined again at 1100 °C for 30 min to oxidize the SiC NFA, and the final product was obtained: a nanofiber aerogel coated with a nanolayer of SiO_2_, named SiC@SiO_2_ NFA.

### Characterization

The microstructures of the SiC@SiO_2_ NFA were examined by scanning electron microscopy (SEM, Apreo 2, Thermo Fisher Scientific, USA) and transmission electron microscopy (TEM, Talos F200X S/TEM, Thermo Fisher Scientific, USA) from Thermo Fisher Scientific. Raman spectra were acquired using a confocal Raman microscope system (Raman, LabRAM HR Evolution, HORIBA Jobin Yvon S.A.S., France). The phase formations of the SiC@SiO_2_ NFA were identified by X-ray diffraction (XRD, Smartlab, Rigaku Corporation, Japan). The chemical structures of the sample were characterized via Fourier transform infrared (FTIR, Nicolet iS10, Thermo Fisher Scientific, USA) and X-ray photoelectron spectrometry (XPS, EscaLab Xi + , Thermo Fisher Scientific, USA). The thermal stability of the SiC@SiO_2_ NFA was characterized by thermogravimetric analysis (TGA, STA 409 PC/4/H, NETZSCH-Gerätebau GmbH, Germany) in an air/Ar atmosphere. N_2_ adsorption and desorption isotherms were obtained using a quantachrome instrument (BSD-PM1/2, Beishide Instrument Technology Co., Ltd., China). The thermal conductivities were measured via the transient hot-wire method (HCDR-S, Nanjing Huicheng Instruments Co. Ltd., China). A contact angle analyzer was used to characterize the hydrophilic/hydrophobic properties of the samples (CA500S, Kunshan Beidou Precision Instrument Co., Ltd., China). The compressive stress of the as-prepared samples was measured by using an electronic universal material testing machine (JHYC, Nanjing Juhang Technology Co., Ltd., China). The piezoresistive response of the materials was recorded using a source meter system (KEITHLEY 6514, Keithley Instruments, Inc., USA), which was linked to a motor unit and a computer. The heat transfer process in the sample was assessed by using an infrared imaging system (FLIR E750, FLIR Systems, USA). A vector network analyzer was used to evaluate the EM parameters of the samples (Agilent N5234A, Keysight Technologies, Inc., USA).

## Results and Discussion

### Preparation Mechanism, Structural and Compositional Characterization of the SiC@SiO_2_ NFA

**Fig. 1:**
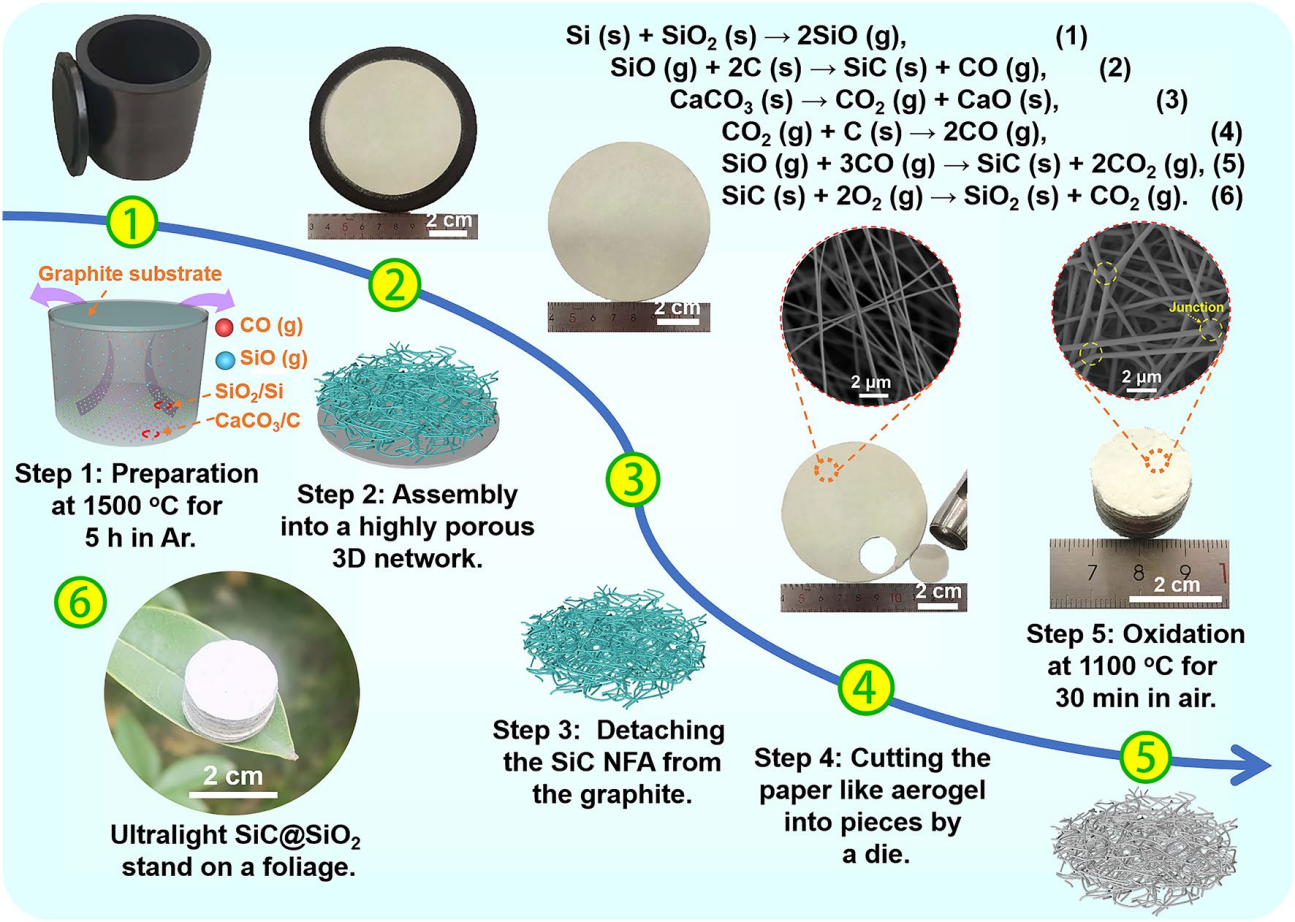
Preparation process for the SiC@SiO_2_ NFA. ① Step 1: Preparation at 1500 °C for 5 h in Ar. ② Step 2: The self-assembly into a 3D highly porous aerogel. ③ Step 3: Detachment of the SiC NFA from the graphite lid at 700 °C for 2 h in air. ④ Step 4: The aerogels are punched into circular tablets with a diameter of 2 cm. ⑤ Oxidation of SiC NFA to form stable cross-linking junctions at 1100 °C for 30 min in air and SiO_2_ coating on each nanofiber, marked as SiC@SiO_2_ NFA. ⑥ The as-prepared SiC@SiO_2_ NFA with ultralow density (~ 11 mg cm^−3^) standing on foliage

As illustrated in Fig. 1, the SiC@SiO_2_ NFA was fabricated via a simple CVD method and subsequent heat treatment process, which was divided into five steps: (1) First, SiO_2_ and Si nanopowder were selected as silicon sources, and CaCO_3_ and activated carbon hybrid particles were selected as carbon sources. Then, these materials were poured into a graphite crucible and calcined at 1500 °C for 5 h in Ar in a nitriding furnace. At high temperature, the Si and SiO_2_ mixed nanoparticles reacted chemically to generate SiO gas (Eq. (1) inserted in Fig. 1). The SiO gas then reacted with the free carbon on the lid of the graphite crucible, providing the formation possibility of the SiC nuclei (Eq. (2) inserted in Fig. 1). As the temperature increased, CaCO_3_ began to decompose to form CO_2_ gas (Eq. (3) inseted in Fig. 1). The CO_2_ gas reacting with the activated carbon led to the CO gas (Eq. (4) inserted in Fig. 1). The large amount of SiO and CO gases generation ensured the continuous growth of SiC nanofibers (Eq. (5) insert in Fig. 1). (2) As the reaction proceeded, the nanofibers continued to nucleate and grow on the surface of the grown nanofibers forming a 3D network. (3) When the reaction was complete, SiC nanofibers were deposited on the surface of the graphite lid, forming a certain thickness of aerogel. Notably, we needed to exfoliate the aerogel from the graphite lid by a calcination process at 700 °C for 5 h in air, labeled as SiC nanofiber aerogel (SiC NFA). (4) The SiC NFA was punched into circular tablets with a diameter of 2 cm. (5) Finally, the as-prepared sample was calcined at 1100 °C for 30 min in air to partially oxidize the surface of the SiC nanofibers. After this treatment, SiC nanofibers coated with SiO_2_ nanolayers were obtained as the product (Eq. (6) inserted in Fig. 1), designated as SiC@SiO_2_ nanofiber aerogel (SiC@SiO_2_ NFA). Moreover, some of the SiC were oxidized and decomposed to generate SiO and CO gases during the oxidation process. These gases would be involved in the regeneration of SiC nanoparticles/nanofibers, which tightly cross-linked the nanofibers together through a large number of junction nodes to form a more stable 3D network structure. Notably, the density of the as-prepared SiC@SiO_2_ NFA obtained by this method was only ~ 11 mg cm^−3^, with a porosity of ~ 99.6% (Table S1), and it is so light that it can even stand on a foliage ((6) inserted in Fig. 1).

**Fig. 2:**
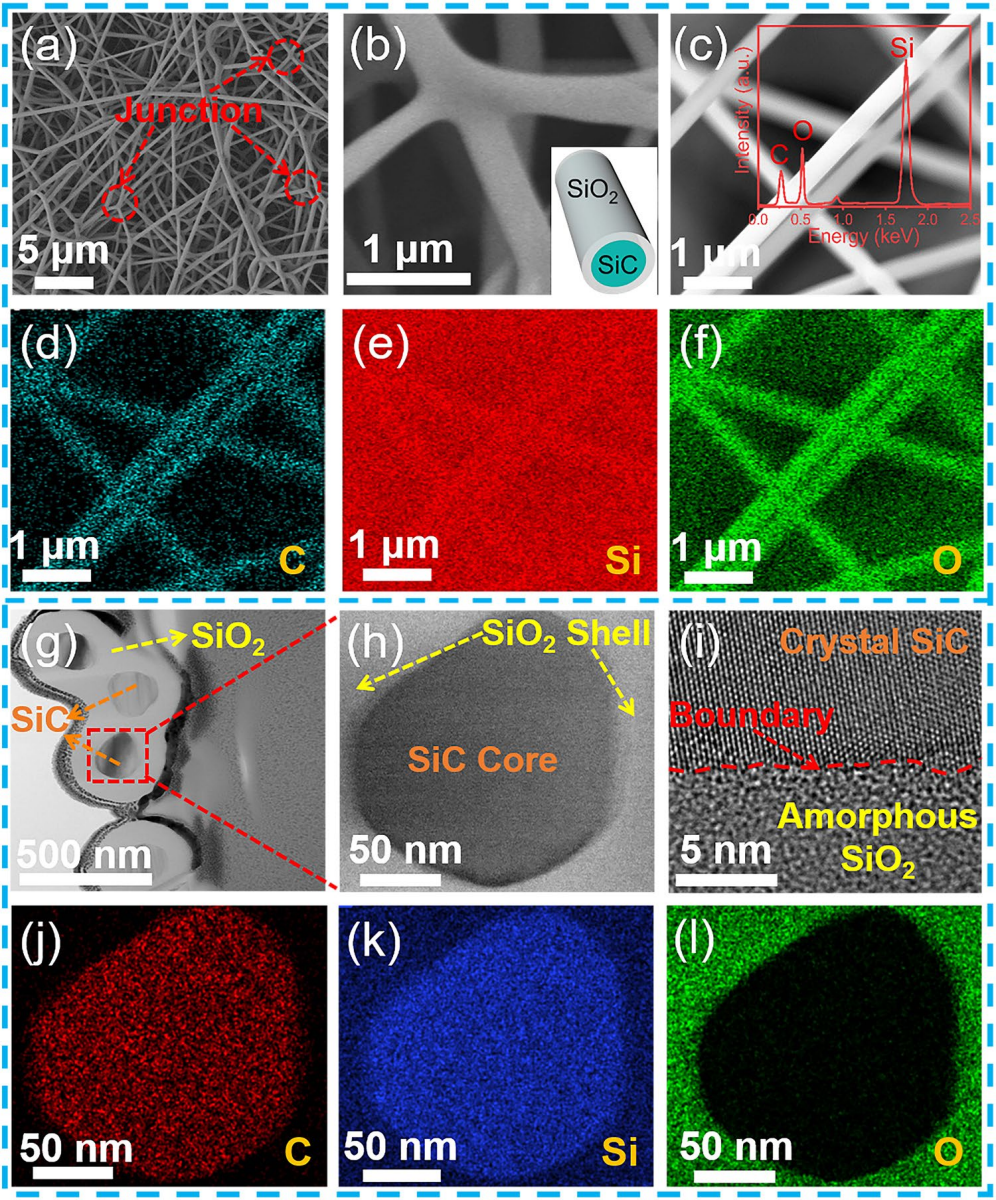
Microscopic structure of the SiC@SiO_2_ NFA. SEM images of **a** line cluster cross-linking microstructure of the SiC@SiO_2_ NFA, **b** three cross-linked SiC@SiO_2_ nanofibers to form a junction and a schematic of a SiC nanofiber coated with a nanolayer of SiO_2_ (inset), **c** the SiC@SiO_2_ nanofibers with a smooth and round surface and the corresponding EDS spectrum for the nanofibers (inset), and **d–f** the corresponding EDS mappings from **c**. TEM images of **g** the SiC@SiO_2_ nanofibers cross section with a core−shell structure, **h** a SiC@SiO_2_ nanofiber cross section at magnification, **i** boundary between the SiC and SiO_2_, and **j–l** the corresponding element mapping for **h**

The microstructures of the SiC@SiO_2_ NFA were characterized by using SEM and TEM. Figure 2a shows the SEM image of the resulting SiC@SiO_2_ NFA, which consisted of cross-linked SiC@SiO_2_ nanofibers. These nanofibers are hundreds of micrometers in length and 200 − 400 nm in diameter and are well interconnected through junction nodes. These fiber-to-fiber junctions are greatly beneficial for promoting the structural integrity and maximizing the mechanical strength. A magnified SEM image of a junction node formed by three SiC@SiO_2_ nanofibers cross-linked together is shown in Fig. 2b, and the illustration further demonstrates its tightly wrapped feature (insert in Fig. 2b). The SiC nanofibers have an angular microstructure with hexagonal prism-like structures (Fig. S1a). The corresponding results of the energy dispersive spectrum (EDS, Fig. S1b) show only signals of C and Si elements from the SiC nanofibers. In contrast, a smooth nanolayer covers the surface of the SiC nanofiber through the oxidation process (Fig. 2c), and the corresponding elemental mapping confirms that the SiC@SiO_2_ nanofibers are mainly composed of C (blue dots), Si (red dots), and O (green dots) elements (Fig. 2d–f). Furthermore, the inset EDS spectrum in Fig. 2c further demonstrates that the nanofibers mainly contain C, Si, and O, confirming the oxidation of SiC nanofibers and the formation of SiO_2_. The TEM image in Fig. 2g shows the cross section of SiC@SiO_2_ nanofiber junction in the SiC@SiO_2_ NFA, and the corresponding cross section preparation process is presented in Fig. S2a–e. Moreover, the core–shell structure composed of SiC and SiO_2_ can be observed. The crystal structure of the SiC core (Fig. 2h) is further characterized using HRTEM and confirmed its 3C-SiC structure, corresponding to the (111) plane with a lattice spacing of 0.25 nm in the image (Fig. S3a)  [36]. The corresponding selected area electron diffraction (SAED) pattern (Fig. S3b) further proves that the SiC core of the SiC@SiO_2_ nanofiber is a single crystal structure  [37]. The boundary between SiC and SiO_2_ is presented in Fig. 2i, and the lattice structure of SiO_2_ on one side of the boundary cannot be observed. Meanwhile, element mapping analysis (Fig. 2j–l) shows the elemental distribution of Si, C, and O elements, which further proves the successfully synthesized SiO_2_ coatings on the SiC nanofiber.

**Fig. 3:**
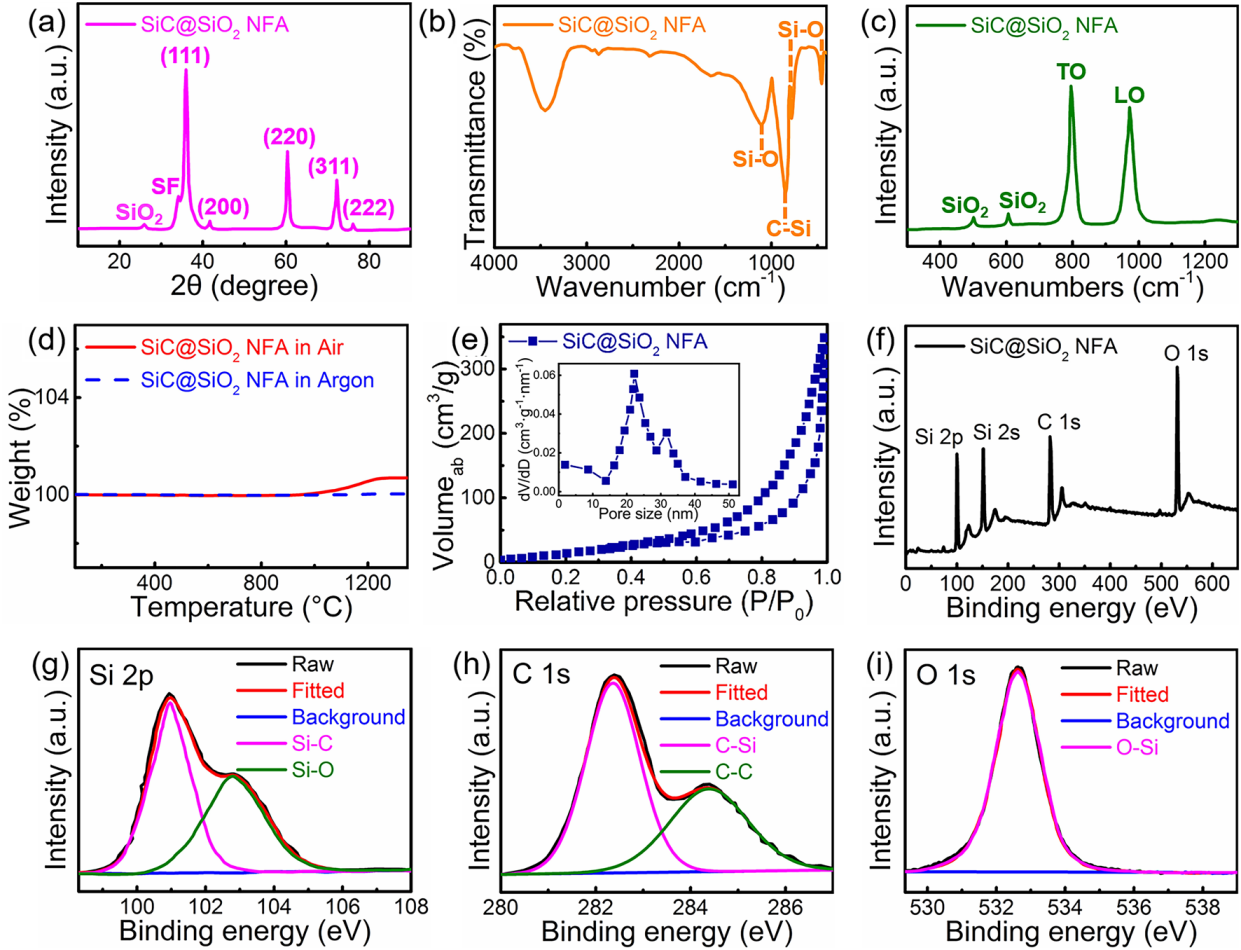
Crystal structure, thermal stability, pore structure, and chemical composition of the SiC@SiO_2_ NFA. **a** XRD patterns, **b** FTIR spectra, **c** Raman spectra, and **d** TGA curves, **e** N_2_ adsorption/desorption isotherms and corresponding adsorption pore size distribution (inset), **f** XPS survey spectrum and high-resolution **g** Si 2*p*, **h** C 1*s*, and **i** O 1*s* XPS spectra for the SiC@SiO_2_ NFA

In Fig. 3a, a typical XRD pattern for the SiC@SiO_2_ NFA is presented. The main diffraction peaks correspond to the (111), (200), (220), (311), and (222) planes of 3C-SiC (JCPDS No. 29–1129) [38]. A weak peak appeared at 33.6° is considered to be the stacking fault of plane (111) [39], and this completely corresponds to the XRD diffraction peaks observed for the SiC NFA (Fig. S4). In addition, a broad peak of silica (SiO_2_) phase appears at 2θ = 26°, due to the partial oxidation of SiC nanofibers during the oxidation process at 1100 °C [40]. The FT-IR spectrum for the SiC@SiO_2_ NFA (Fig. 3b) is compared with that of the SiC NFA (Fig. S5); not only Si–C bonds are observed at approximately 838 cm^−1^, but also Si–O absorption bands appear at 1095, 779, and 455 cm^–1^ [41]. There are two characteristic peaks at 795 and 972 cm^− 1^ in the Raman spectrum of the SiC@SiO_2_ NFA (Fig. 3c), corresponding to that observed for the SiC NFA (Fig. S6), which are related to the transversal optic (TO) and longitudinal optic (LO) modes of Si–C vibrations at the Γ point, respectively [42]. Besides, the peaks at 499 and 605 cm^− 1^ are assigned to the bending motion and stretching vibration of the Si – O bond, respectively [43]. It should be mentioned that the Raman effect of SiO_2_ is much weaker than that of SiC due to its amorphous structure. The TG curves measured from 25 to 1350 °C are used to evaluate the mass changes of the SiC@SiO_2_ NFA in air and in argon atmosphere (Fig. 3d). The results show a straight line under the argon atmosphere, indicating that no chemical reaction occurs. Moreover, the sample weight only increased by 0.69% in air. This result indicates that the SiO_2_ nanolayer formed during the oxidation process can effectively slow the inward diffusion of oxygen in the nanofibers thus protecting the nanofibers from further oxidation. Figure 3e presents the N_2_ adsorption/desorption isotherm and BJH pore size distributions of the SiC@SiO_2_ NFA. In the relative pressure range of 0.5 – 1.0, the curve presents a type-IV isotherm with an obvious capillary condensation phenomenon, showing that the aerogel has a 3D mesoporous network structure [44]. The BET specific surface area of the SiC@SiO_2_ NFA is 185.3 m^2^ g^− 1^ while the pore size of the SiC@SiO_2_ NFA is distributed at approximately 22 nm (Fig. 3e, insert), and the corresponding results are listed in Table S1. The elements in the samples were scanned with high resolution by an XPS analyzer, and the chemical information on the surface of the samples was analyzed. As shown in Fig. 3f, the XPS survey spectra show the signals of C, Si, and O elements, which is in consistence with the EDS results. The Si 2*p* peak (Fig. 3g) reveals two peaks at 101 and 102.8 eV due to Si–C and Si–O bonds, respectively [45]. As presented in Fig. 3h, there are two peaks at 282.3 and 284.4 eV in the C 1*s* spectrum, corresponding to the C–Si and C–C bonds, respectively. For the high-resolution scans for O 1*s* (Fig. 3i), a characteristic peak located at 532.6 eV is observed, which is associated with the O – Si bond of the SiO_2_ nanolayer.

### Superelasticity of the SiC@SiO_2_ NFA under Severe Temperature Variations

**Fig. 4:**
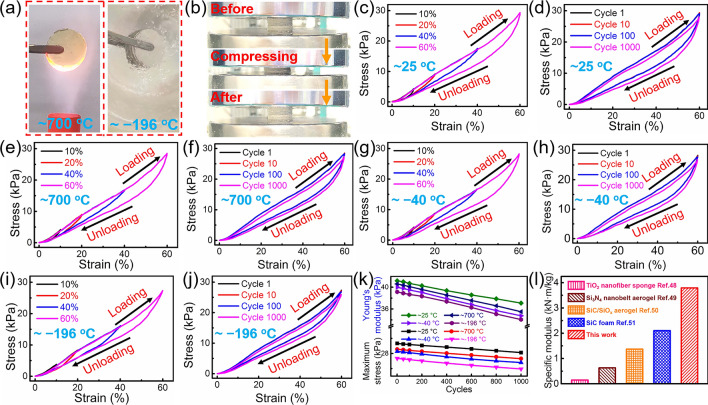
Temperature-invariance hyperelasticity of the SiC@SiO_2_ NFA. **a** The SiC@SiO_2_ NFA placed inside the flame of an alcohol blowtorch (~ 700 °C) and immersed in liquid nitrogen (~ − 196 °C). **b** Compression test for the SiC@SiO_2_ NFA, which can quickly recover to its original shape. Compression stress–strain curves for the SiC@SiO_2_ NFA at **c** ~ 25 °C, **e** ~ 700 °C, **g** ~  − 40 °C and **i** ~  − 196 °C. Cyclic compression stress–strain curves for the SiC@SiO_2_ NFA at **d** ~ 25 °C, **f** ~ 700 °C, **h** ~  − 40 °C and **j** ~  − 196 °C. **k** The maximum stress and Young’s modulus as a function of the compression test cycles. **l** Comparison of the specific modulus of the SiC@SiO_2_ NFA with that of other aerogels with random structures

The elastic property of the materials plays a key role in high-level EMW absorption and piezoresistivity and pressure sensing applications. Although the SiC@SiO_2_ NFA is heat-treated at different temperatures, it is still able to maintain its macrostructural integrity, as shown in Figs. 4a and S7. The SiC@SiO_2_ NFA can be easily compressed into thin sheets, which then return to their original shape when the pressure was released, demonstrating its excellent mechanical properties that allows for large compression deformations without structural failure (Fig. 4b). In detail, Fig. 4c shows the stress–strain curves at 10%, 20%, 40%, and 60% strains, and the SiC@SiO_2_ NFA recovers its original configuration even after 60% strain at ~ 25 °C. The loading process for the SiC@SiO_2_ NFA also exhibits deformation stages similar to those reported for nanofibrous aerogels [46]: linear elastic deformation state (ε < 20%) and a nonlinear regime with a steep increase in stress (*ε* > 20%). This resilient compressibility is further highlighted by a durable cyclic performance at 60% strain with a high strain rate of 80 mm min^− 1^ (Fig. 4d). The SiC@SiO_2_ NFA possesses a ceramic nature and nanofiber microstructures, which are expected to achieve stable superelasticity at various extreme service temperatures. First, the hyperelasticity of the SiC@SiO_2_ NFA at high temperature is confirmed by compression testing after heat treatment with an alcohol torch. As expected, the stress–strain curves after high-temperature treatment show a deformation trend similar to that found for original SiC@SiO_2_ NFA (~ 700 °C, Fig. 4e). The heat-treated aerogel also endures 1000 fatigue cycles at 60% strain with a strain rate of 80 mm/min (Fig. 4f). This high speed in the loading–unloading cycles demonstrates the superb elastic recovery ability of the SiC@SiO_2_ NFA. After 1000 cycles, the SiC@SiO_2_ NFA almost retains its original macroscopic shape completely with only a slight permanent deformation. Then, the SiC@SiO_2_ NFA was placed on an iron plate above a jar filled with liquid nitrogen to measure its superelasticity at low temperature (~ − 40 °C). The stress–strain curves obtained for the SiC@SiO_2_ NFA (Fig. 4g) at 10%, 20%, 40%, and 60% strain are similar to those at room temperature, indicating that the SiC@SiO_2_ NFA still has excellent mechanical properties after low-temperature treatment. The aerogel also exhibits good cyclic fatigue resistance (strain rate: 80 mm/min), as displayed in Fig. 4h. Finally, the SiC@SiO_2_ NFA also shows excellent robust hyperelasticity at ultralow temperatures (~ − 196 °C) under direct immersion in liquid nitrogen. The stress–strain curves obtained at different strains (Fig. 4i) and 1000 compression cycles test (Fig. 4j) for the SiC@SiO_2_ NFA show that there is not much difference compared to the test results obtained at ~  − 40 °C.

The maximum stress and Young’s modulus during cyclic compression for the SiC@SiO_2_ NFA treated at various temperatures are shown in Fig. 4k and Table S2. For the first cycle, the maximum stress and Young’s modulus at ~ 25 °C are 29.33 and 41.17 kPa, respectively. After 1000 cycles, the maximum stress and Young’s modulus are 28.12 and 37.08 kPa, respectively. It can be observed that both the maximum compressive strength and Young’s modulus show only a slight decrease, indicating that the SiC@SiO_2_ NFA has a nearly constant compressive strength at ~ 25 °C. Likewise, the values for the maximum compressive strength and the Young’s modulus also show only a small drop after 1000 cycles at ~ 700, ~  − 40, and ~  − 196 °C, respectively. These results are similar to those obtained for the SiC@SiO_2_ NFA at ~ 25 °C. In addition, the Young’s modulus (*E*) of the aerogel at ~ 25 °C is approximately 41.17 kPa. The calculated specific modulus (E/ρ) is ~ 3.74 kN m kg^−1^, which is significantly higher than that of other work (Fig. 4l) [47–50]. These highlight that the present SiC@SiO_2_ NFA has excellent mechanical properties.

### Piezoresistivity and Pressure Sensing Properties of the SiC@SiO_2_ NFA for Detecting Human Motions

**Fig. 5:**
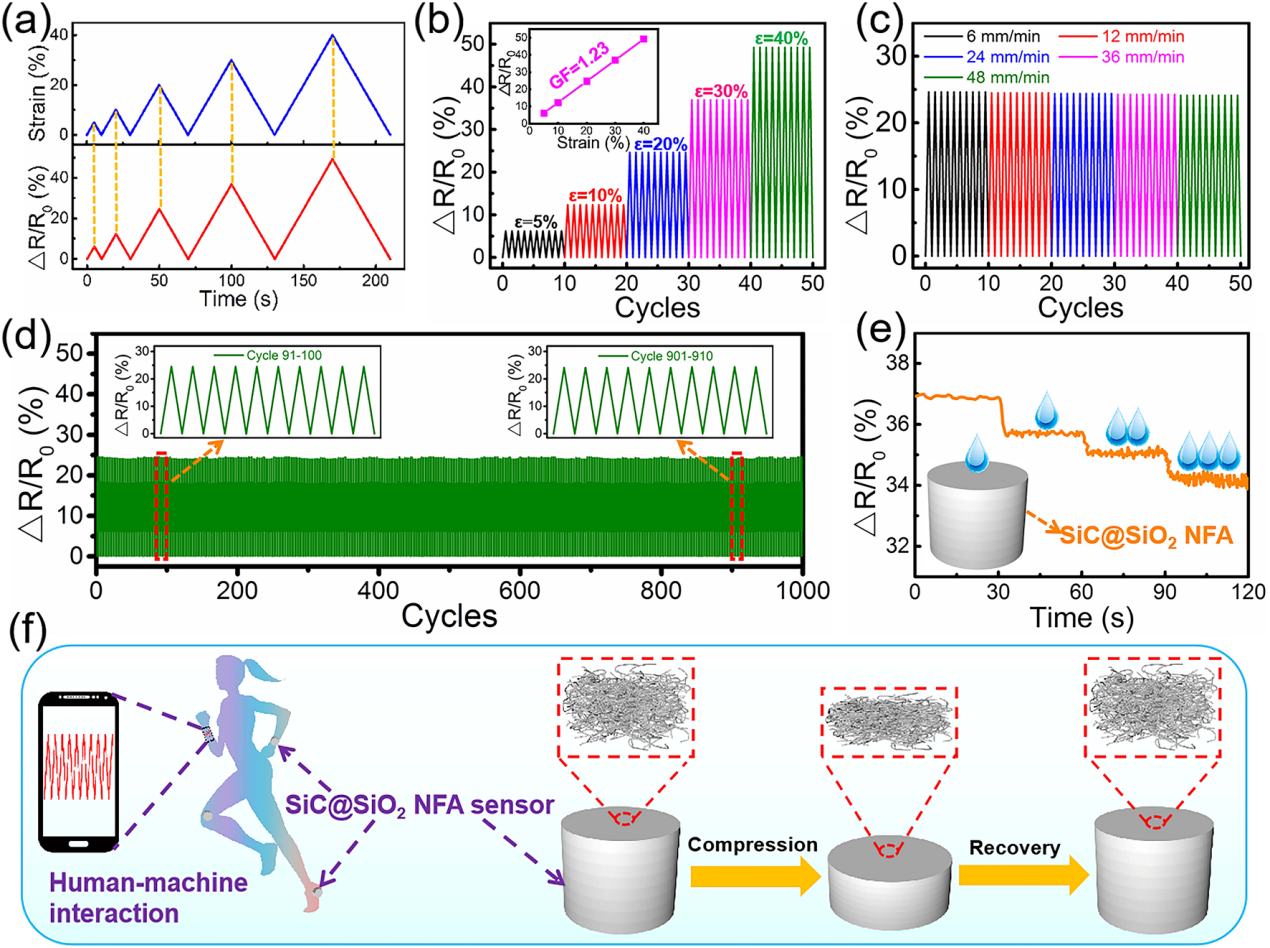
Strain- and pressure-sensing behaviors of the SiC@SiO_2_ NFA. **a** Δ*R*/*R*_0_ for the SiC@SiO_2_ NFA with the strain from 5 to 40% at a compression rate of 6 mm min^−1^. **b** Real-time Δ*R*/*R*_0_ cycling test at different compression strains under a compression speed of 6 mm/min; Δ*R*/*R*_0_ varies linearly with strain (inset **b**, GF = 1.23). **c** Δ*R*/*R*_0_ for the SiC@SiO_2_ NFA under various compression rates with a compression strain of 30%. **d** Stability testing of the piezoresistive behavior of SiC@SiO_2_ NFA with a 30% compressive strain, 6 mm min^−1^ compression rate, and 1000 cycles (inset shows the magnified curves). **e** Real-time ΔR/R_0_ response in the presence of drops of NaCl aqueous solution (inset depicts the corresponding schematic diagram for the NaCl aqueous solution drop tests). **f** Applications of the SiC@SiO_2_ NFA pressure sensor to detect body activities and tiny pressures

The SiC cores of the SiC@SiO_2_ nanofibers are semiconducting, and their resistance value will change accordingly with compression deformation; hence, a SiC@SiO_2_ NFA-based piezoresistive pressure sensor is fabricated. Figure 5a displays the change in resistance (Δ*R*/*R*_0_ = (*R*_0_ − *R*)/*R*_0_ (7) [51], where *R*_0_ and *R* represent the incipient resistance and momentary resistance, respectively) of the SiC@SiO_2_ NFA for a gradual increase in strain from 5 to 40% at a compression rate of 6 mm min^−1^. The Δ*R*/*R*_0_ increases proportionally with the strain, suggesting that the SiC@SiO_2_ NFA possesses remarkable strain-dependent piezoresistive sensing behavior. As shown in Fig. 5b, Δ*R*/*R*_0_ varies under different cyclic strains. The resistance can completely return to its initial value owing to the excellent compressive recoverability and fatigue resistance, which exhibits outstanding strain-sensing reversibility for every stage. Furthermore, the change in the relative resistance and the increase in compression strain show a clear linear relationship, yielding a gauge factor (GF = (Δ*R*/*R*_0_)/*ε* (8)) of 1.23 (inset Fig. 5b). This result demonstrates the huge potential of using the SiC@SiO_2_ NFA in sensors with excellent repeatability to detect various strains. Meanwhile, the dependence on the speed of the external compression was also examined. Figure 5c exhibits the Δ*R*/*R*_0_ for the piezoresistive pressure sensor at varying compression rates of 6, 12, 24, 36, and 48 mm min^− 1^. The various compression rates have little effect on the maximum ΔR/R_0_ value under the same strain of 30%, which is crucial for the stability of the sensor in practical applications. It is noteworthy that the resistance variation ratio of the SiC@SiO_2_ NFA sensor for 30% strain with a compression rate of 6 mm min^−1^ shows no noticeable change after 1000 compression cycles (Fig. 5d), and the inset displays no obvious attenuation, which originates from the excellent superelasticity and microstructural reversibility of the SiC@SiO_2_ NFA. In addition, the sensor can be exposed to the moisture in the air in practical applications; in particular, it will inevitably come cross human sweat in the process of detecting human motion. Therefore, it is particularly important to simulate the effect of human sweat on the sensor resistance. As shown in Fig. 5e, Δ*R*/*R*_0_ decreases stably as an aqueous solution of NaCl is gradually dripped onto the sensor. The possible reason for this is that the penetration of NaCl solution into the interior of the SiC@SiO_2_ NFA increases its electrical conductivity, thereby reducing the resistance. Therefore, the amount of human motion can be effectively detected according to the real-time change in the resistance to ensure the health of the human body. Figure 5f shows a schematic of the SiC@SiO_2_ NFA sensor used to detect human motion and the mechanism of the piezoresistive sensing performance. In practical applications, pressure sensors can be used to detect human activity and tiny pressures and then transmit this information to mobile phones. The principle of realizing this function is that SiC@SiO_2_ NFA is compressed and bent upon loading, which leads to the nanofibers touching and interlinking with neighboring nanofibers. A large number of temporary junction contacts shorten the transport path for electrons through the aerogel, thereby reducing the electrical resistance [52]. These contacts disappear after unloading, and the resistance fully returns to its original value. It is believed that the superb structural integrity and compressive recoverability of the SiC@SiO_2_ NFA dictate the piezoresistivity of the sensing behavior.

### Application as a Super Thermal Insulator at Extreme Temperatures

**Fig. 6:**
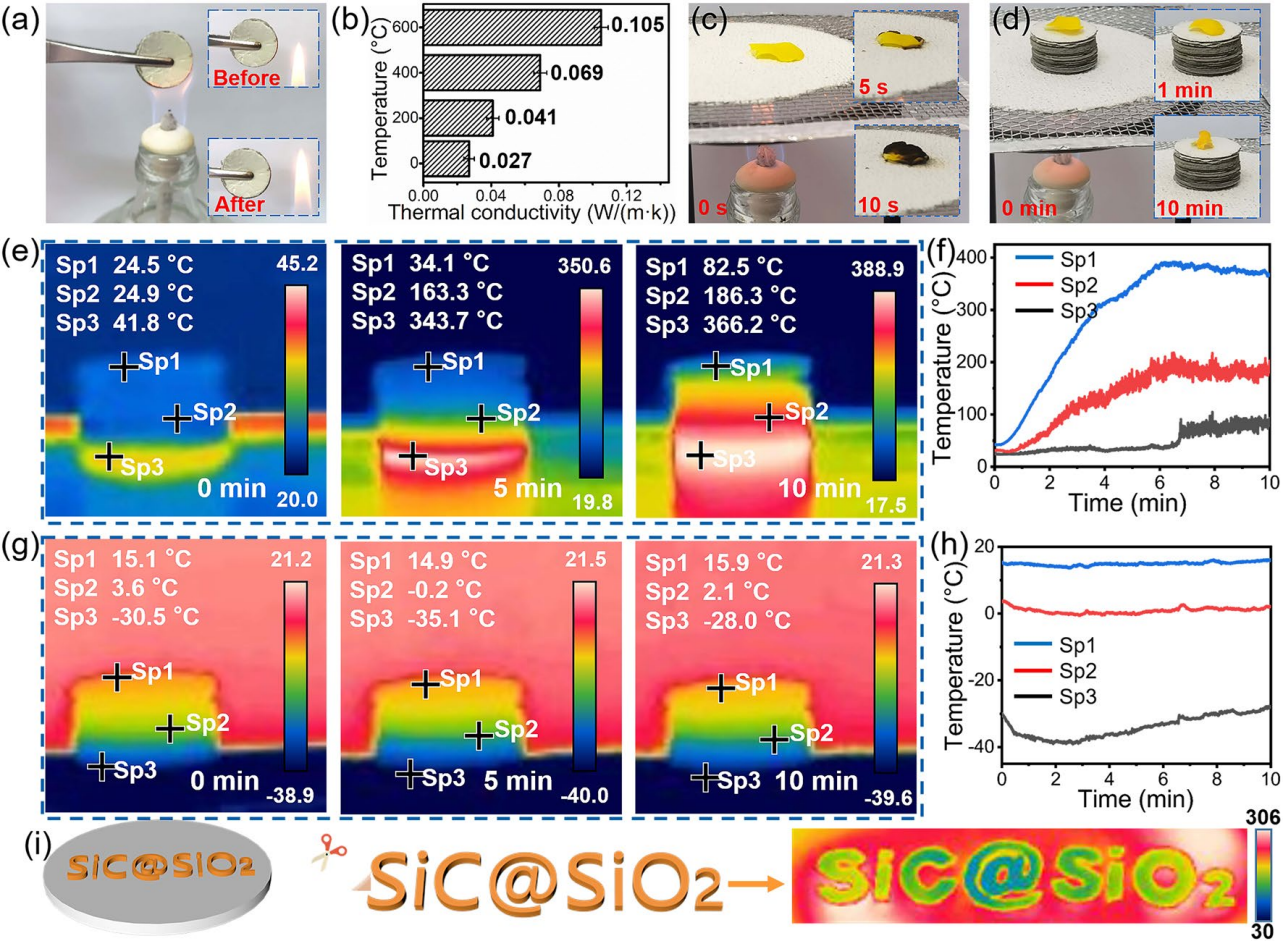
Fire and high/low-temperature resistance and thermal insulation performance of the SiC@SiO_2_ NFA. **a** Digital photographs of the SiC@SiO_2_ NFA exposed to the flame of an alcohol lamp. **b** Thermal conductivities of the SiC@SiO_2_ NFA at various temperatures in an argon atmosphere. **c** A flower placed onto the asbestos network and **d** a flower placed onto the SiC@SiO_2_ NFA in a burner flame. Thermal images of the SiC NFAS recorded during **e** heating on a heated platform and **g** freezing on a refrigeration platform with the corresponding (**f** and **h**) temperature vs. time curves. **i** Schematic of the thermal insulation mechanism for the SiC@SiO_2_ NFA

The as-prepared SiC@SiO_2_ NFA exhibits excellent chemical and thermal stability at high temperature, which is crucial for high-temperature EMW-absorbing applications. As shown in Fig. 6a, the macroscopic shape of the SiC@SiO_2_ NFA does not change when the aerogel is placed in the alcohol flame for 10 min, indicating that it has superb ablation resistance and thermal stability. The temperature-dependent thermal conductivities of the SiC@SiO_2_ NFA in an argon atmosphere are presented in Fig. 6b and Table S3. Notably, the thermal conductivity of the SiC@SiO_2_ NFA at room temperature is only 0.027 W m^−1^ K^−1^, suggesting that the obtained aerogel is an excellent thermal insulator. The thermal conductivity increases with increasing temperature from 20 to 600 °C, which is mainly related to thermal radiation at high temperature [53]. As shown in Fig. 6c, a flower is carbonized within 10 s when it is placed onto a heated asbestos mesh. However, the fresh flower can survive after 10 min heating when being placed on a piece of aerogel (thickness, 10 mm, Fig. 6d). This result further proves that the SiC@SiO_2_ NFA has excellent thermal insulation properties. Figure 6e shows the real-time temperature measured from the side of the SiC@SiO_2_ NFA on a heating platform. After 10 min, the temperatures at the top (Sp1), middle (Sp2), and bottom (Sp3) are 82.5, 186.3, and 366.2 °C, respectively. The temperature at the top is much lower than that at the bottom and middle and reaches a relatively stable value of ~ 82 °C after 7 min under the same heating conditions. The corresponding real-time temperatures are shown in Fig. 6f. Additionally, Fig. 6g shows the real-time temperatures measured for the SiC@SiO_2_ NFA on a refrigeration platform. The corresponding temperatures at the top (Sp1), middle (Sp2), and bottom (Sp3) are 15.9, 2.1, and − 28 °C after 10 min, respectively. It can be observed that the temperature at the top is much higher than that at the bottom and middle, as evident from the corresponding real-time temperature vs. time graph (Fig. 6h). This result indicates that SiC@SiO_2_ NFA also has excellent thermal insulation properties at low temperature. The SiC@SiO_2_ NFA can also be cut into various shapes owing to its superior flexibility. The letters “SiC@SiO_2_” cut from the SiC@SiO_2_ NFA are clearly visible in the thermal image against a heated platform (~ 306 °C), as shown in Fig. 6i, suggesting that SiC@SiO_2_ NFA has great potential for blocking infrared signal transmissions. The thermal insulation mechanism mainly involves two aspects: (1) when heat flux is transferred in the 3D network structure of the SiC@SiO_2_ NFA, the limited contact surface among the SiC@SiO_2_ nanofibers can effectively reduce the solid-phase heat conduction; (2) the SiC@SiO_2_ NFA possesses a large number of mesoporous structures, which can shackle air molecules to decrease the gas-phase thermal convection. These results also confirm that SiC@SiO_2_ NFA possesses outstanding thermal insulation properties, therefore, it can be used as a potential high-performance thermal insulation material in the aerospace field.

### High Absorption Capacities and Self-cleaning Properties of the Oil-modified SiC@SiO_2_ NFA

**Fig. 7:**
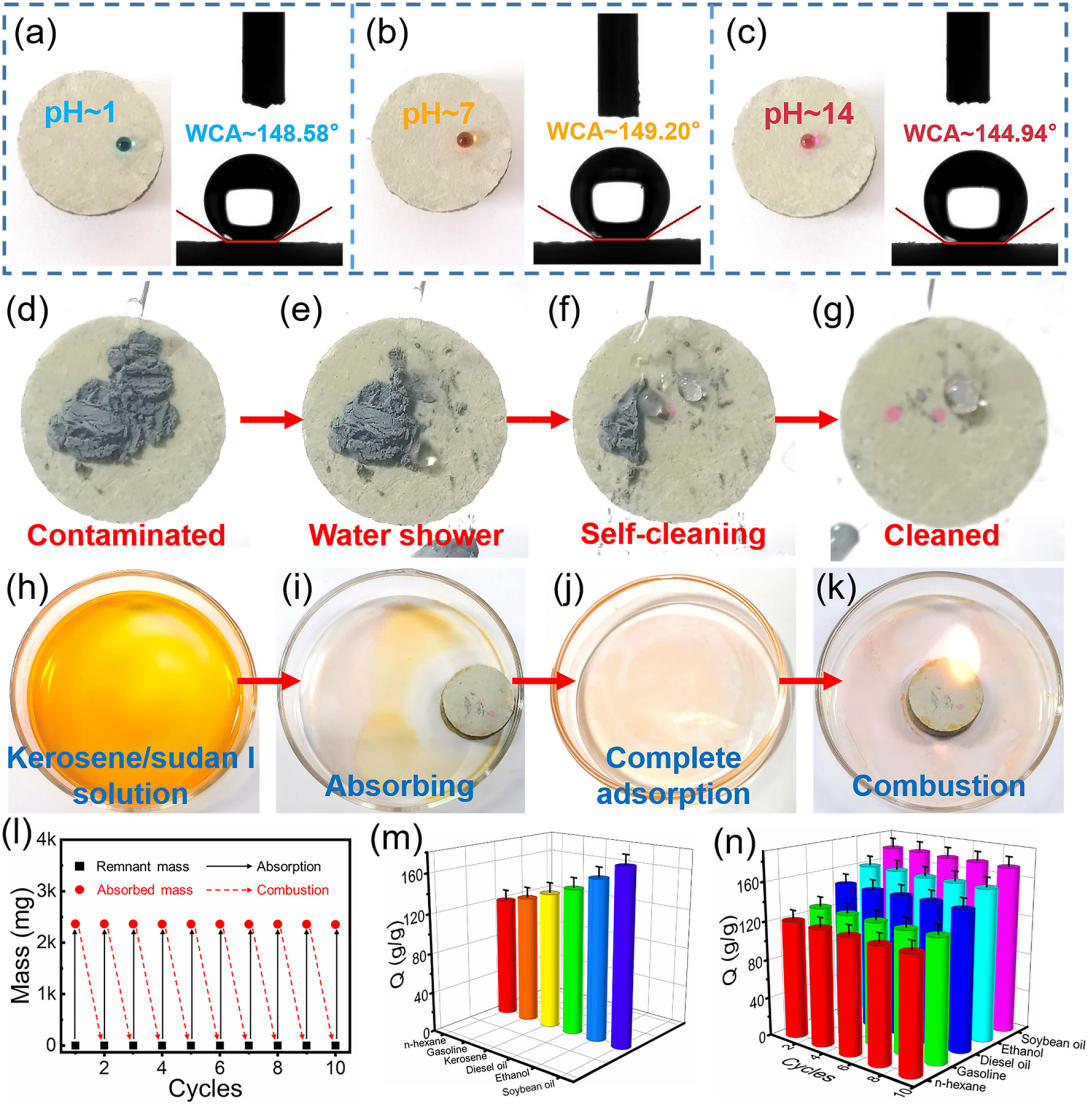
High absorption capacities for organic liquids and the self-cleaning property of the oil-modified SiC@SiO_2_ NFA. **a − c** Digital photograph of a water droplet with pH value ~ 1, ~ 7, and ~ 14 on an oil-modified SiC@SiO_2_ NFA and the corresponding WCA images, respectively. **d–g** Self-cleaning process of the oil-modified SiC@SiO_2_ NFA. **h–k** Process of absorption of methyl orange aqueous solution by the oil-modified SiC@SiO_2_ NFA and a subsequent combustion test. **l** Recyclability of the oil-modified SiC@SiO_2_ NFA in the absorption of kerosene by the combustion method. **m** Absorption capacities of the oil-modified SiC@SiO_2_ NFA for various organic liquids and the corresponding **n** recyclability of the SiC@SiO_2_ NFA

The pristine SiC@SiO_2_ NFA is superhydrophilic with a water contact angle (WCA) of ~ 0°, as displayed in Fig. S8. Hydrophilic SiC@SiO_2_ NFA can be converted to a hydrophobic material by oil impregnation of the surface of the aerogel [54]. In Fig. 7a, a blue acidic water droplet at pH = 1 displays a WCA value of ~ 148.58° on the surface of a piece of the oil-modified SiC@SiO_2_ NFA. At pH = 7, the orange color water droplet is recorded with a WCA value of ~ 149.2° (Fig. 7b). Similarly, the rose-red alkaline water droplet still has a spherical morphology on the surface of the oil-modified SiC@SiO_2_ NFA, which exhibits a slightly lower WCA value (~ 144.94°) than the other two water droplets, as shown in Fig. 7c. These results demonstrate the excellent hydrophobicity of the oil-modified SiC@SiO_2_ NFA in various solutions, which lays the foundation for investigating the self-cleaning property of the prepared aerogel. To confirm the self-cleaning performance of the obtained aerogel, silicon nanopowders were dispersed across the surface of the oil-modified SiC@SiO_2_ NFA. Then, a 2.5 mL syringe was used to flush a piece of dusty sample surface with water droplets to confirm the self-cleaning performance of the obtained aerogel. As shown in Fig. 7d–g, the powder-laden surface is well cleaned by the water droplet. To further expand the practicality of the oil-modified SiC@SiO_2_ NFA aerogels in terms of hydrophobicity, their adsorption of organic solvents and oils was investigated. As demonstrated in Fig. 7h–j, a kerosene/Sudan I solution was quickly absorbed, which can also be consumed by combustion (Fig. 7k).

Cycling experiments with absorbing and burning organic solvents were carried out to further evaluate the cycling stability of the oil-modified SiC@SiO_2_ NFA. As displayed in Fig. 7I, black colour cycles of the combustion test were performed in an air atmosphere, and no obvious adsorption capacity change is found during this process. In addition, the aerogel experienced no change throughout the burning test, which exhibits good flame retardancy and a robust structure. The practicability of the oil-modified SiC@SiO_2_ NFA is further explored, as revealed in Fig. 7m. Specifically, the broad applicability of the oil-modified SiC@SiO_2_ NFA for adsorbing organic solvents was verified by adsorbing n-hexane, gasoline, kerosene, diesel, ethanol, and soybean oil. The results show that the absorption weight of these organic solvents is equivalent to 121 to 175 times the weight of the oil-modified SiC@SiO_2_ NFA, which depends on the surface tension and density of the adsorbed organic liquid. Additionally, these absorbed organic liquids were all subjected to 10-cycle combustion tests, as shown in Fig. 7n. The oil-modified SiC@SiO_2_ NFA retains its original structure and appearance during these combustion cycle tests, further demonstrating the excellent three-dimensional structural stability and ablation resistance of the aerogel. These results indicate that oil-modified SiC@SiO_2_ NFA can be used as a highly efficient selective adsorption material. In addition, the excellent hydrophobicity of oil-modified SiC@SiO_2_ NFA is the key to its use as a high-performance EMW-absorbing material.

### EMW Absorption Performance of the SiC@SiO_2_ NFA

Reflection loss (RL) is an important factor to evaluate the EMW absorption property of the SiC@SiO_2_ NFA, which can be calculated by as follows [55, 56]:

1 $${\text{RL}} = 20\lg \left| {\frac{{Z_{{{\text{in}}}} - Z_{o} }}{{Z_{{{\text{in}}}} + Z_{o} }}} \right|$$

2 $$Z_{in} = Z_{o} \sqrt {\frac{{\mu_{r} }}{{\varepsilon_{r} }}} \tanh \left[ {j\frac{{2\pi fd\sqrt {\mu_{r} \varepsilon_{r} } }}{c}} \right]$$

where $Z_{in}$ is the input impedance of the aerogel, *f* is the frequency, *c* is the speed of light, d is the thickness of the aerogel, and $Z_{o}$ is the impedance in free space.

**Fig. 8:**
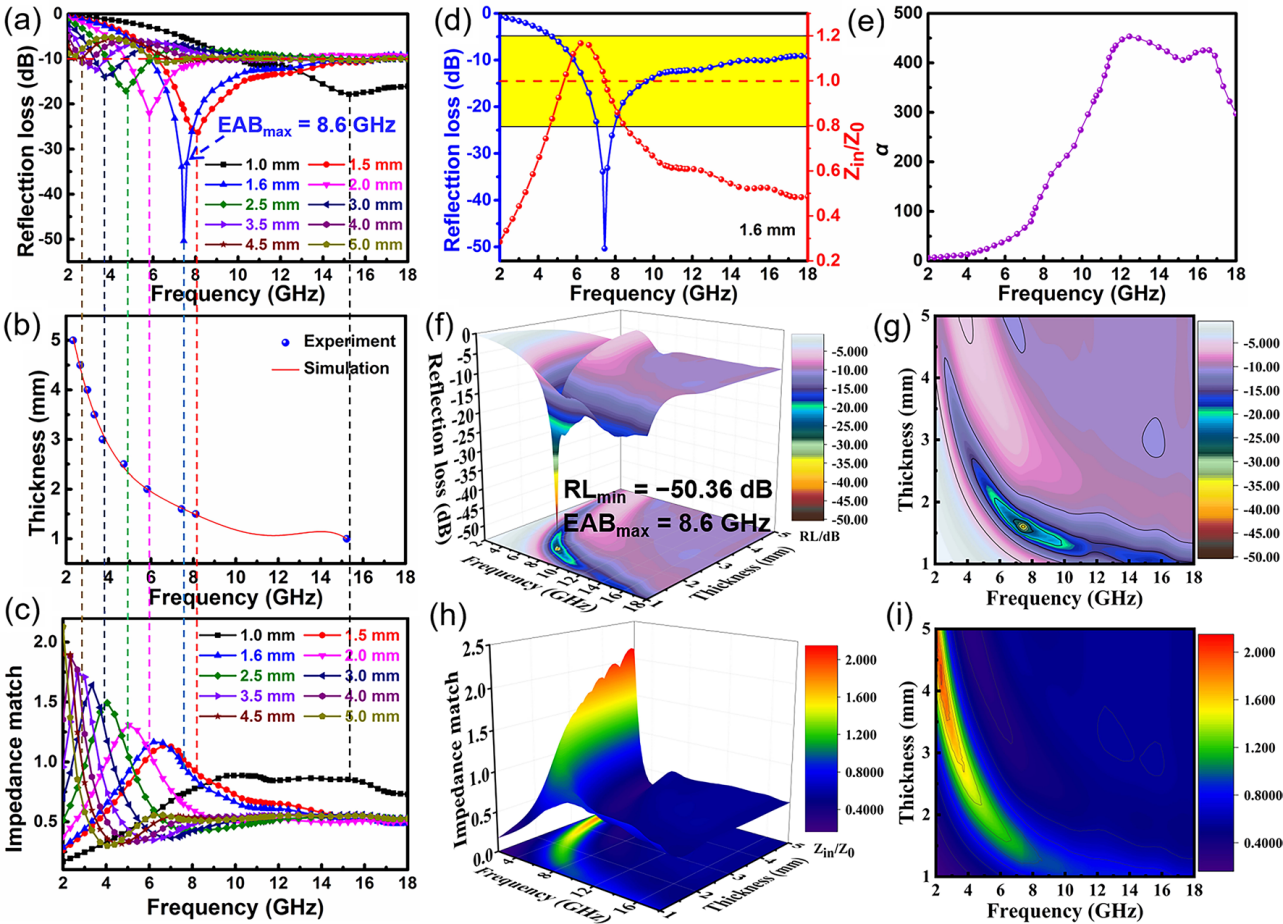
EMW absorption performance of the SiC@SiO_2_ NFA. **a** Frequency- and thickness-dependent RL values, **b** the relationship between the simulation thickness and peak RL at typical frequencies, **c** the frequency- and thickness-dependent impedance matching (Z), **d** the relationship between *RL*_min_ and Z at a thickness of 1.6 mm, **e** the attenuation constant α, **f** 3D and **g** 2D representations, and **h** 3D and **i** 2D plots of Z for the SiC@SiO_2_ NFA

We know that a *RL* value of − 10 dB means 90% absorbed of the incident EMW radiation, and the corresponding bandwidth indicates an effective absorption bandwidth (EAB) [57, 58]. As shown in Fig. 8a and Table S1, the *EAB*_max_ for the SiC@SiO_2_ NFA is 8.6 GHz corresponding to a frequency range of 5.82–14.42 GHz, while the RL_min_ value for the SiC@SiO_2_ NFA is − 50.36 dB at 7.44 GHz (thickness, 1.6 mm). According to the quarter-wave attenuation law [59], the *RL*_min_ value shifts to low frequencies with the increasing thickness (Fig. 8b):

3 $$t_{m} = n\lambda /4 = \frac{nc}{{4f_{m} \sqrt {\left| {\mu_{r} } \right|\left| {\varepsilon_{r} } \right|} }} \left( {n = 1, 3, 5...} \right)$$

Optimization of the impedance matching (|$Z_{in}$/$Z_{o}$|) results in excellent EMW absorption performance. The value |$Z_{in}$/$Z_{o}$| of 1 indicates the absorber has a great impedance match and let EMW easily enter inside. Figure 8c shows that the value of |$Z_{in}$/$Z_{o}$| is close to 1 for the SiC@SiO_2_ NFA in the thickness range of 1.6–2 mm, indicating that the incident EMW can effectively enter the interior of the aerogel, which can be converted into heat to be consumed to avoid reflection into the air at the interface. In Fig. 8d, it further proves that a good impedance matching can be beneficial to the improving EMW absorption property of the materials. The other influence factor of attenuation constant (*α*) can be calculated from Eq. (4) [60, 61]:

4 $$\alpha = \frac{\sqrt 2 \pi f}{c} \times \sqrt {\left( {\mu^{\prime\prime}\varepsilon^{\prime\prime} - \mu^{\prime}\varepsilon^{\prime}} \right) + \sqrt {\left( {\mu^{\prime}\varepsilon^{\prime\prime} + \mu^{\prime\prime}\varepsilon^{\prime}} \right)^{2} + \left( {\mu^{\prime\prime}\varepsilon^{\prime\prime} - \mu^{\prime}\varepsilon^{\prime}} \right)^{2} } }$$

It is generally believed that the larger α value is, the greater ability of the absorber attenuates the EMWs, as displayed in Fig. 8e. Moreover, 3D plots and 2D RL diagrams for the SiC@SiO2 NFA with various thicknesses at 2–18 GHz are shown in Fig. 8f–g. The 3D and 2D diagrams for the Z of the SiC@SiO_2_ NFA corresponding to the thickness and frequency are further shown in Fig. 8h–i. The Z values for the SiC@SiO_2_ NFA with a thickness of 1 ~ 2 mm almost always range between 0.8 and 1.2 at frequencies from 4 to 9 GHz. The results demonstrate the superb impedance matching of the aerogel can be an important factor for the excellent EMW-absorbing properties.

**Fig. 9:**
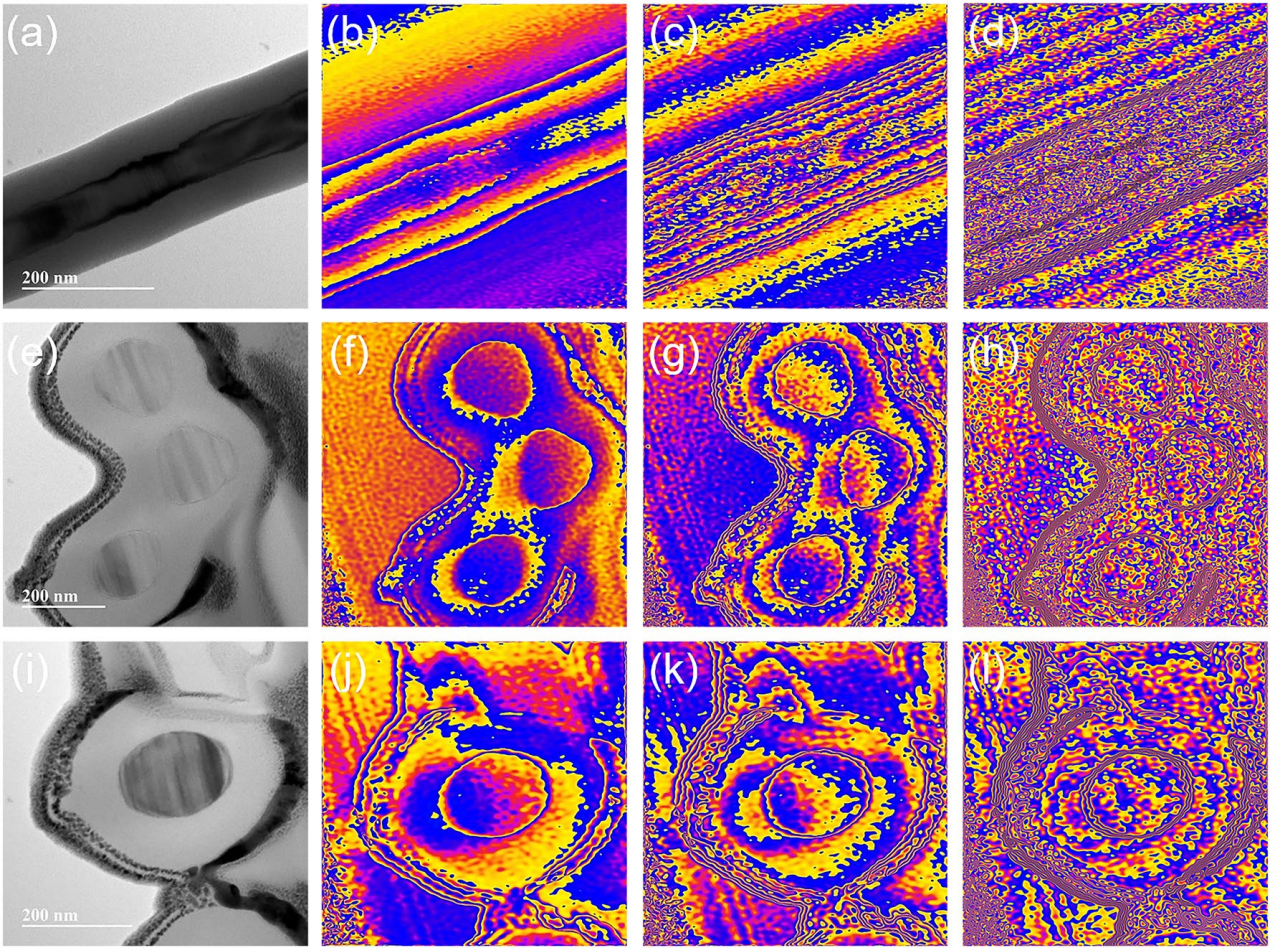
Off-axis electron holography images of the SiC@SiO_2_ NFA. **a** TEM image and **b–d** charge density images of the longitudinal section of a SiC@SiO_2_ nanofiber. **e** TEM image and **f–h** charge density images of the transverse section of a junction node. **i** TEM image and **j–l** charge density images of the transverse section of a SiC@SiO_2_ nanofiber

Off-axis electron holographic analysis can clearly reveal the dielectric polarization, especially the potential orientation and charge density distribution at specific interface regions, which can be characterized intuitively and quantitatively [62]. Figure 9a–d shows the TEM image and corresponding charge density images under various amplified signals obtained for the longitudinal section of a SiC@SiO_2_ nanofiber. It can be clearly observed that the charges are concentrated at the SiC/SiO_2_ and SiO_2_/air interfaces with the continuous amplification of the signal, resulting in a strong interface polarization. Furthermore, the two ends of each SiC@SiO_2_ nanofiber easily form induced polarized charges during the propagation of EMWs inside the aerogel owing to the large aspect ratio of the nanofibers. Therefore, each nanofiber can be regarded as a one-dimensional vibrating electric dipole and generate periodic motions to dissipate the EMW energy under alternating EM fields [63]. In addition to the dipolar polarization, the heterostructure of the SiC@SiO_2_ nanofibers also contributes significantly to the permittivity enhancement. As shown in Fig. 9e, three fibers are cross-linked together to form a junction node (TEM image of the transverse section). The free charges can be trapped at these nodes originating from the difference in Fermi levels, which is essentially due to the various dielectric constant properties of SiC and SiO_2_ [64]. Figure 9f–h displays the state of the charge density distribution at the interface between SiC and SiO_2_ to form an obvious local polarization field with increasing signal intensity, which will greatly consume the incident EMWs and enhance the microwave absorption property. In addition, it can be clearly observed from Fig. 9i that SiC and SiO_2_ grow closely together and make it possible for the leakage and tunneling of electrons. The charges accumulated at the interface (Fig. 9j–l) could break the potential barrier under a local strong electric field, which expands the electron transition path and further dissipates the energy of the EMW. The charge density distribution in the SiC core of the SiC@SiO_2_ nanofibers further confirms that the 3D cross-linked aerogels can be used as an electron transport network, thus contributing to a conductive loss.

**Fig. 10:**
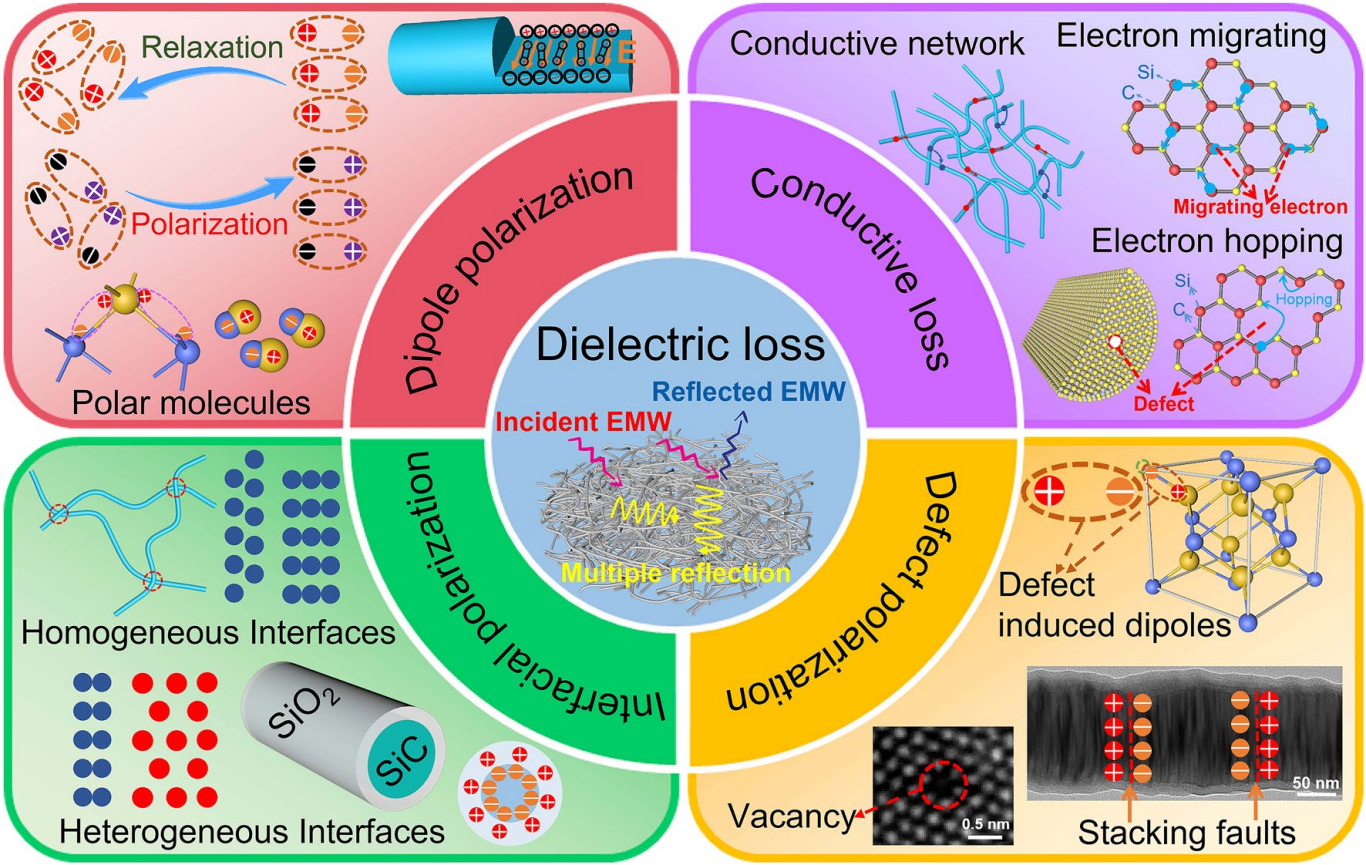
Schematic of the EMW absorption mechanisms for the SiC@SiO_2_ NFA

Actually, SiC is an excellent dielectric loss electromagnetic wave absorption material [57, 65], and SiO_2_ is an electromagnetic wave transparent material [66, 67]. When the electromagnetic wave is incident on the surface of SiC@SiO_2_ nanofiber, the SiO_2_ nanolayer can lock the electromagnetic wave to avoid being reflected, and the SiC core can effectively convert electromagnetic energy into heat or electricity energy. These results suggest that the synergistic effect of the SiC cores and SiO_2_ nanolayer of the SiC@SiO_2_ nanofiber enables aerogel to exhibit excellent electromagnetic wave absorption properties. Herein, we comprehensively studied the EMW-absorbing mechanisms for the SiC@SiO_2_ NFA from a perspective of dielectric loss, including multiple reflection, conduction loss, defect-induced polarization, interfacial polarization, and dipolar polarization, and the results are shown in Fig. 10.

The SiC@SiO_2_ NFA was constructed by a large number of cross-linked SiC@SiO_2_ nanofibers, which have a 3D porous structure. The incident EMW was attenuated by multiple reflections among the pores, resulting in the conversion of EM energy into heat for dissipation [68]. The conduction loss was caused by the converted energy of the EMW into an electric current when it propagated in the SiC@SiO_2_ nanofibers. When the generated current was transported along the nanofibers, Joule heat was generated due to the resistance of the SiC@SiO_2_ nanofibers, which consumed the EMW energy [69].

In general, electron migration and electron hopping are two common types of conductive loss models. The 3D network structure constructed from SiC@SiO_2_ nanofibers shows enhanced electrical conductivity; under an external EM field, and the electrons will flow along the radial direction of nanofibers and rapidly propagate out to the entire 3D network [70]. In other words, electron migration simply refers to the free movement of electrons in the process of propagation. Moreover, electron hopping mainly refers to the transfer of electrons among the SiC@SiO_2_ nanofibers [71]. However, the SiO_2_ shell on the nanofiber surface is nonconductive, and the high energy barrier will greatly limit the electron hopping process.

It is worth noting that defects can also lead to electron hopping, which also involves another dielectric loss mechanism—defect polarization. It can be determined that oxygen vacancies are introduced during the oxidation process to form defect sites in the SiC@SiO_2_ nanofibers. The charge carriers can be trapped at these defect sites, leading to an imbalanced charge distribution. The resulted polarization and EM energy loss then occur [72]. Furthermore, 2H-SiC fragments are embedded in the 3C-SiC grains during the formation of 3C-SiC nanofibers, and the resulting 3C/2H-SiC heterostructures form stacking faults [73]. Charge separation easily occurs at the interface of stacking faults, which induces the generation of dipoles and increases the polarization loss of dipoles.

The interface polarization effect is also known as the Maxwell–Wagner-Sillars effect [74]. The enhancement of the interface polarization effect can improve the dielectric loss capacity, thereby promoting electromagnetic wave loss. The unique core–shell structure of SiC@SiO_2_ nanofibers contributes to the interface polarization. As discussed before, in situ growth of a layer of wave-transparent SiO_2_ on the surface of SiC nanofibers cannot only achieve a good impedance matching but also form a heterogeneous interface at the connection between SiC and SiO_2_ in the SiC@SiO_2_ NFA. The accumulated charges and collective interface polarization can lead to the conversion of EM energy into heat [75]. Therefore, the interfacial polarization and dipole relaxation induced by the SiO_2_ nanolayers can improve the dielectric loss. In addition, a local strong electric field is generated due to the difference in electrical conductivity between the SiC and SiO_2_ [76]. When the electric field strength is sufficient to breakdown the SiO_2_ nanolayer, electrons will pass freely among the SiC@SiO_2_ nanofibers, thereby forming homogeneous interfaces. Predictably, the SiC@SiO_2_ nanofibers are well interconnected by junction nodes (Fig. 2b), and the SiC cores at the junction also form homogeneous interfaces.

It is well known that dipole polarization involves the movement of polar or nonpolar molecules under a changing electromagnetic field. As a polar molecule, the inherent dipole rearrangement of SiC will occur under the action of an external EM field, so it is called directional polarization [77]. The SiC cores of the SiC@SiO_2_ nanofibers undergo dipole polarization and relaxation processes under a change in the EM field, consuming the EM energy. In addition, transverse electric fields are formed inside the SiC@SiO_2_ nanofibers under the action of the dipoles, and the electrons are affected by the transverse electric field during the movement process, which increases the transmission path and further consumes the EM energy. Thus, the synergistic effect of multiple reflection, conduction loss, defect-induced polarization, interfacial polarization, and dipolar polarization together enables the excellent EMW-absorbing property of the SiC@SiO_2_ NFA.

## Conclusions

We have successfully fabricated 3D porous cross-linked SiC@SiO_2_ NFAs by combining a simple CVD technique with a subsequent heat treatment process. The obtained aerogel displays outstanding properties, including an ultralow density (~ 11 mg cm^−3^), thermal superinsulation (0.027 W m^−1^ K^−1^), great recoverable compressibility (repeated full recovery from 60% strain), good thermal and chemical stabilities, and significant strain-dependent piezoresistive sensing behavior. Furthermore, the oil-modified SiC@SiO_2_ NFA exhibits superb hydrophobicity and self-cleaning feature, which can adsorb a great quantity of organic liquids (121–175 times its own weight). The SiC@SiO_2_ NFA also shows excellent EMW-absorption performance, with a remarkable *RL*_min_ of − 50.36 dB at 7.44 GHz and thickness of 1.6 mm, and a superwide *EAB* of 8.6 GHz over the frequency range of 5.82–14.42 GHz. Given the excellent multifunctional properties of this material, we believe it has great potentials for various practical applications in areas such as elastic components, high-efficiency oil/water adsorption materials, piezoresistive pressure sensors, and high-performance EMW absorbers in extreme environments.

## Supplementary Information

Below is the link to the electronic supplementary material.

Supplementary file1 (PDF 660 KB)

## References

[1] Li G, Dong D, Hong G, Yan L, Zhang X (2019). High efficiency cryo-thermocells assembled with anisotropic holey graphene aerogel electrodes and a eutectic redox electrolyte. Adv. Mater..

[2] Xu X, Zhang Q, Hao M, Hu Y, Lin Z (2019). Double-negative-index ceramic aerogels for thermal superinsulation. Science.

[3] Chhowalla M, Jariwala D (2019). Hyperbolic 3D architectures with 2D ceramics. Science.

[4] Yu Z, Qin B, Ma Z, Huang J, Li S (2019). Superelastic hard carbon nanofiber aerogels. Adv. Mater..

[5] Pierre AC, Pajonk GM (2002). Chemistry of aerogels and their applications. Chem. Rev..

[6] Zhang LS, Tang ZC, Tusiime R, Wang SF, Feng NN (2021). Synthesis and electromagnetic wave absorbing properties of a polymer-derived SiBNC ceramic aerogel. Ceram. Int..

[7] Zhao WY, Shao G, Jiang MJ, Zhao B, Wang HL (2017). Ultralight polymer-derived ceramic aerogels with wide bandwidth and effective electromagnetic absorption properties. J. Eur. Ceram. Soc..

[8] Kumar R, Sahoo S, Joanni E, Singh RK, Tan WK (2021). Recent progress on carbon-based composite materials for microwave electromagnetic interference shielding. Carbon.

[9] Kumar R, Macedo WC, Singh RK, Tiwari VS, Constantino CJL (2019). Nitrogen-sulfur Co-doped reduced graphene oxide-nickel oxide nanoparticle composites for electromagnetic interference shielding. ACS Appl. Nano Mater..

[10] Kumar R, Alaferdov AV, Singh RK, Singh AK, Shah J (2019). Self-assembled nanostructures of 3D hierarchical faceted-iron oxide containing vertical carbon nanotubes on reduced graphene oxide hybrids for enhanced electromagnetic interface shielding. Compos. Part B Eng..

[11] Leventis N, Sadekar A, Chandrasekaran N, Sotiriou-Leventis C (2010). Click synthesis of monolithic silicon carbide aerogels from polyacrylonitrile-coated 3D silica networks. Chem. Mater..

[12] Lin Z, Zeng Z, Gui X, Tang Z, Zou M (2016). Carbon nanotube sponges, aerogels, and hierarchical composites: synthesis, properties, and energy applications. Adv. Energy Mater..

[13] Jiang S, Agarwal S, Greiner A (2017). Low-density open cellular sponges as functional materials. Angew. Chem. Int. Ed..

[14] Si Y, Wang X, Yan C, Yang L, Yu J (2016). Ultralight biomass-derived carbonaceous nanofibrous aerogels with super-elasticity and high pressure-sensitivity. Adv. Mater..

[15] Ziegler C, Wolf A, Liu W, Herrmann AK, Gaponik N (2017). Modern inorganic aerogels. Angew. Chem. Int. Ed..

[16] Cai J, Liu S, Feng J, Kimura S, Wada M (2012). Cellulose-silica nanocomposite aerogels by in situ formation of silica in cellulose gel. Angew. Chem. Int. Ed..

[17] Verdolotti L, Lavorgna M, Lamanna R, Maio ED, Iannace S (2015). Polyurethane-silica hybrid foam by sol-gel approach: chemical and functional properties. Polymer.

[18] Qiu L, Liu JZ, Chang SLY, Wu Y, Li D (2012). Biomimetic superelastic graphene-based cellular monoliths. Nat. Commun..

[19] Si Y, Yu J, Tang X, Ge J, Ding B (2014). Ultralight nanofibre-assembled cellular aerogels with superelasticity and multifunctionality. Nat. Commun..

[20] Fang XS, Bando Y, Gautam UK, Ye CH, Golberg D (2008). Inorganic semiconductor nanostructures and their field-emission applications. J. Mater. Chem..

[21] Hu P, Dong S, Zhang XH, Gui KH, Chen GQ (2017). Synthesis and characterization of ultralong SiC nanowires with unique optical properties, excellent thermal stability and flexible nanomechanical properties. Sci. Rep..

[22] Nakamura D, Gunjishima I, Yamaguchi S, Ito T, Okamoto A (2004). Ultrahigh-quality silicon carbide single crystals. Nature.

[23] Chen XY, Liu XH, Geng XJ, Jia QL (2018). Photoluminescence properties of SiC/SiO_2_ heterojunctions obtained by TiO_2_-assisted chemical vapor deposition. Ceram. Int..

[24] Khalid B, Bai X, Wei H, Huang Y, Wu H (2017). Direct blow-spinning of nanofibers on a window screen for highly efficient PM2.5 removal. Nano Lett..

[25] Si Y, Zhang Z, Wu W, Fu Q, Huang K (2018). Daylight-driven rechargeable antibacterial and antiviral nanofibrous membranes for bioprotective applications. Sci. Adv..

[26] Wang X, Yu J, Sun G, Ding B (2016). Electrospun nanofibrous materials: a versatile medium for effective oil/water separation. Mater. Today.

[27] Lu D, Su L, Wang HJ, Niu M, Xu L (2019). Scalable fabrication of resilient SiC nanowires aerogels with exceptional high-temperature stability. ACS Appl. Mater. Interfaces.

[28] Dong YY, Zhu XJ, Pan F, Cai L, Jiang HJ (2022). Implanting NiCo_2_O_4_ equalizer with designable nanostructures in agaric aerogel-derived composites for efficient multiband electromagnetic wave absorption. Carbon.

[29] Huang WH, Qiu Q, Yang XF, Zuo SW, Bai JA (2022). Ultrahigh density of atomic CoFe-electron synergy in noncontinuous carbon matrix for highly efficient magnetic wave adsorption. Nano Micro Lett..

[30] Zhao YP, Zuo XQ, Guo Y, Huang H, Zhang H (2021). Structural engineering of hierarchical aerogels comprised of multi-dimensional gradient carbon nanoarchitectures for highly efficient microwave absorption. Nano Micro Lett..

[31] Wu SS, Fu H, Hu XS, Ding CY, Yan X (2022). High aspect-ratio sycamore biomass microtube constructed permittivity adjustable ultralight microwave absorbent. J. Colloid Interface Sci..

[32] Jang HK, Kim J, Park JS, Moon JB, Oh J (2022). Synthesis and characterization of a conductive polymer blend based on PEDOT:PSS and its electromagnetic applications. Polymers.

[33] Malere CPR, Donati B, Eras N, Silva VA, Lona LF (2021). Electromagnetic evaluation of radar absorbing materials based on conducting polypyrrole and organic-inorganic nanocomposite of polypyrrole/kaolinite. J. Appl. Polym. Sci..

[34] Gong YB, Yang ZG, Wei XG, Song SL, Ma SQ (2022). Synthesis and electromagnetic wave absorbing properties of high-entropy metal diboride-silicon carbide composite powders. J. Mater. Sci..

[35] Fan YT, Yang D, Mei H, Xiao SS, Yao YT (2022). Tuning SiC nanowires interphase to improve the mechanical and electromagnetic wave absorption properties of SiCf/SiCnw/Si_3_N_4_ composites. J. Alloy. Compd..

[36] Wu RB, Zhou K, Yang ZH, Qian XK, Wei J (2013). Molten-salt-mediated synthesis of SiC nanowires for microwave absorption applications. CrystEngComm.

[37] Cheng YH, Tan MY, Pi Hu, Zhang XH, Sun BQ (2018). Strong and thermostable SiC nanowires/graphene aerogel with enhanced hydrophobicity and electromagnetic wave absorption property. Appl. Surf. Sci..

[38] Han M, Yin X, Duan W, Ren S, Zhang L (2016). Hierarchical graphene/SiC nanowire networks in polymer-derived ceramics with enhanced electromagnetic wave absorbing capability. J. Eur. Ceram. Soc..

[39] Chen J, Shi Q, Tang W (2011). Field emission performance of SiC nanowires directly grown on graphite substrate. Mater. Chem. Phys..

[40] An ZM, Ye CS, Zhang RB, Zhou P (2019). Flexible and recoverable SiC nanofiber aerogels for electromagnetic wave absorption. Ceram. Int..

[41] Su L, Wang HJ, Niu M, Dai S, Cai ZX (2020). Anisotropic and hierarchical SiC@SiO_2_ nanowire aerogel with exceptional stiffness and stability for thermal superinsulation. Sci. Adv..

[42] Bechelany M, Brioude A, Cornu D, Ferro G, Miele P (2007). A Raman spectroscopy study of individual SiC nanowires. Adv. Funct. Mater..

[43] Wang CL, Fang ZW, Yi AL, Yang BC, Wang Z (2021). High-Q microresonators on 4H-silicon-carbide-on-insulator platform for nonlinear photonics. Light Sci. Appl..

[44] Zu G, Shimizu T, Kanamori K, Zhu Y, Maeno A (2018). Transparent, superflexible doubly cross-linked polyvinylpolymethylsiloxane aerogel superinsulators via ambient pressure drying. ACS Nano.

[45] Peng K, Zhou JX, Gao HF, Wang JW, Wang HJ (2020). Emerging one-/two-dimensional heteronanostructure integrating SiC nanowires with MoS_2_ nanosheets for efficient electrocatalytic hydrogen evolution. ACS Appl. Mater. Interfaces.

[46] Liang HW, Guan QF, Chen LF, Zhu Z, Zhang WJ (2012). Macroscopic-scale template synthesis of robust carbonaceous nanofiber hydrogels and aerogels and their applications. Angew. Chem. Int. Ed..

[47] Wang HL, Zhang X, Wang N, Li Y, Feng X (2017). Ultralight, scalable, and high-temperature–resilient ceramic nanofiber sponges. Sci. Adv..

[48] Su L, Li MZ, Wang HJ, Niu M, Lu D (2019). Resilient Si_3_N_4_ nanobelt aerogel as fire-resistant and electromagnetic wave-transparent thermal insulator. ACS Appl. Mater. Interfaces.

[49] Ren B, Liu JJ, Rong YD, Wang L, Lu YJ (2019). Nanofibrous aerogel bulk assembled by cross-linked SiC/SiO_x_ core−shell nanofibers with multifunctionality and temperature-invariant hyperelasticity. ACS Nano.

[50] Chabi S, Rocha VG, Garcia-Tunon E, Ferraro C, Saiz E (2016). Ultralight, strong, three-dimensional SiC structures. ACS Nano.

[51] Wang L, Zhang MY, Yang B, Tan JJ, Ding XY (2020). Highly compressible, thermally stable, light-weight, and robust aramid nanofibers/Ti_3_AlC_2_ MXene composite aerogel for sensitive pressure sensor. ACS Nano.

[52] Chen Y, Ola O, Chen HM, Wang NN, Xia YD (2019). SiC nanowire sponges as electropressure sensors. ACS Appl. Nano Mater..

[53] Liang CY, Wang ZF, Wu L, Zhang XC, Wang H (2017). Light and strong hierarchical porous SiC foam for efficient electromagnetic interference shielding and thermal insulation at elevated temperatures. ACS Appl. Mater. Interfaces.

[54] Su L, Wang HJ, Niu M, Fan XY, Ma MB (2018). Ultralight, recoverable, and high-temperature-resistant SiC nanowire aerogel. ACS Nano.

[55] Wang Y, Gao X, Fu Y, Wu X, Wang Q (2019). Enhanced microwave absorption performances of polyaniline/graphene aerogel by covalent bonding. Compos. Part B Eng..

[56] Du H, Zhang QP, Zhao B, Marken F, Gao QC (2021). Novel hierarchical structure of MoS_2_/TiO_2_/Ti_3_C_2_T*x* composites for dramatically enhanced electromagnetic absorbing properties. J. Adv. Cream..

[57] Huang B, Wang ZQ, Hu HL, Tang XZ, Huang XZ (2020). Enhancement of the microwave absorption properties of PyC-SiCf/SiC composites by electrophoretic deposition of SiC nanowires on SiC fibers. Ceram. Int..

[58] Zhang KL, Zhang JY, Hou ZL, Bi S, Zhao QL (2019). Multifunctional broadband microwave absorption of flexible graphene composites. Carbon.

[59] Cheng HR, Pan YM, Wang X, Liu CT, Shen CY (2022). Ni flower/MXene-melamine foam derived 3D magnetic/conductive networks for ultra-efficient microwave absorption and infrared stealth. Nano Micro Lett..

[60] Ding D, Wang Y, Li XD, Qiang R, Xu P (2017). Rational design of core-shell Co@C microspheres for high-performance microwave absorption. Carbon.

[61] Xue W, Yang G, Bi S, Zhang JY, Hou ZL (2021). Construction of caterpillar-like hierarchically structured Co/MnO/CNTs derived from MnO_2_/ZIF-8@ZIF-67 for electromagnetic wave absorption. Carbon.

[62] You W, Che R (2018). Excellent NiO-Ni nanoplate microwave absorber via pinning effect of antiferromagnetic-ferromagnetic interface. ACS Appl. Mater. Interfaces.

[63] Shi X, Yuan J, Zhou W, Rong J, Cao M (2007). Preparation and dielectric properties of nanostructured ZnO whiskers. Chin. Phys. Lett..

[64] Li X, Wang L, You WB, Xing LS, Yang LT (2019). Enhanced polarization from flexible hierarchical MnO_2_ arrays on cotton cloth with excellent microwave absorption. Nanoscale.

[65] Wang CS, Wu SQ, Li ZQ, Chen S, Chen AN (2022). 3D printed porous biomass-derived SiCnw/SiC composite for structure-function integrated electromagnetic aoborption. Virtual Phys. Prototy..

[66] Yuan XY, Cheng LF, Zhang LT (2016). Electromagnetic wave absorbing properties of SiC/SiO_2_ composites with ordered inter-filled structure. J. Alloy. Compd..

[67] Li ZJ, Wang XH, Ling HL, Lin H, Wang T (2020). Electromagnetic wave absorption properties of SiC@SiO_2_ nanoparticles fabricated by a catalyst-free precursor pyrolysis method. J. Alloy. Compd..

[68] Wang CH, Ding YJ, Yuan Y, He XD, Wu ST (2015). Graphene aerogel composites derived from recycled cigarette filters for electromagnetic wave absorption. J. Mater. Chem. C.

[69] Liang C, Qiu H, Han Y, Gu H, Song P (2019). Superior electromagnetic interference shielding 3D graphene nanoplatelets/reduced graphene oxide foam/epoxy nanocomposites with high thermal conductivity. J. Mater. Chem. C.

[70] Wen B, Cao M, Hou Z, Song W, Zhang L (2013). Temperature dependent microwave attenuation behavior for carbon-nanotube/silica composites. Carbon.

[71] Zhu LY, Zeng XJ, Chen M, Yu RH (2017). Controllable permittivity in 3D Fe_3_O_4_/CNTs network for remarkable microwave absorption performances. RSC Adv..

[72] Liu PB, Gao S, Zhang GZ, Huang Y, You WB (2021). Hollow engineering to Co@N-doped carbon nanocages via synergistic protecting-etching strategy for ultrahigh microwave absorption. Adv. Funct. Mater..

[73] Zhang H, Xu Y, Zhou J, Jiao J, Chen Y (2015). Stacking fault and unoccupied densities of state dependence of electromagnetic wave absorption in SiC nanowires. J. Mater. Chem. C.

[74] Qin M, Zhang LM, Wu HJ (2020). Dual-template hydrothermal synthesis of multi-channel porous NiCo_2_O_4_ hollow spheres as high-performance electromagnetic wave absorber. Appl. Surf. Sci..

[75] Xia T, Zhang C, Oyler NA, Chen X (2013). Hydrogenated TiO_2_ nanocrystals: a novel microwave absorbing material. Adv. Mater..

[76] Kuriakose M, Longuemart S, Depriester M, Delenclos S, Sahraoui AH (2014). Maxwell-Wagner-Sillars effects on the thermal-transport properties of polymer-dispersed liquid crystals. Phys. Rev. E.

[77] Li M, Yin X, Zheng G, Chen M, Tao M (2014). High-temperature dielectric and microwave absorption properties of Si_3_N_4_-SiC/SiO_2_ composite ceramics. J. Mater. Sci..

