# Supplementary material for: Multifunctional SiC@SiO2 Nanofiber Aerogel with Ultrabroadband Electromagnetic Wave Absorption
Source: Nanomicro Lett. 2022 Jul 28;14:152. doi: 10.1007/s40820-022-00905-6 (PMC9334492; doi:10.1007/s40820-022-00905-6)
Supplement: Supplementary file 1 — Supplementary file1 (PDF 660 KB) [file 40820_2022_905_MOESM1_ESM.pdf]

Supporting Information for

Multifunctional $SiC@SiO_2$ Nanofiber Aerogel with Ultrabroadband Electromagnetic Wave Absorption

Limeng Song[1], Fan Zhang[1], Yongqiang Chen[1,] *, Li Guan[2], Yanqiu Zhu[3], Mao Chen[1], Hailong Wang[1], Budi Riza Putra[4], Rui Zhang[1,] *, Bingbing Fan[1, 5,] *

[1]School of Materials Science and Engineering, Zhengzhou University, Henan 450001, P. R. China

[2]School of Materials Science and Engineering, Zhengzhou University of Aeronautics, Henan 450015, P. R. China

[3]College of Engineering, Mathematics and Physical Sciences, University of Exeter, Exeter, EX4 4SB, United Kingdom

[4]Research Center for Metallurgy, National Research and Innovation Agency, 15315, Banten, Indonesia

[5]State Key Laboratory of Crystal Materials, Shandong University, Shandong 250100, P. R. China

*Corresponding authors. E-mail: fanbingbing@zzu.edu.cn (Bingbing Fan), chenyq@zzu.edu.cn (Yongqiang Chen), zhangray@zzu.edu.cn (Rui Zhang)

Supplementary Figures and Tables

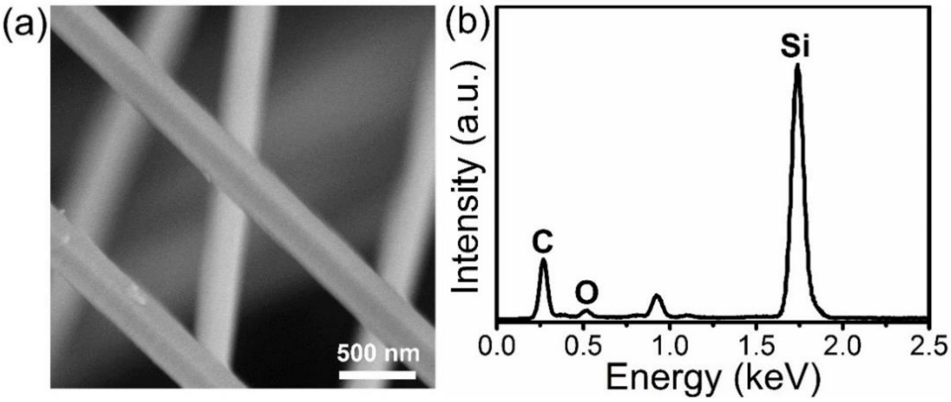

Fig. S1 SEM image of SiC nanofibers and corresponding EDS

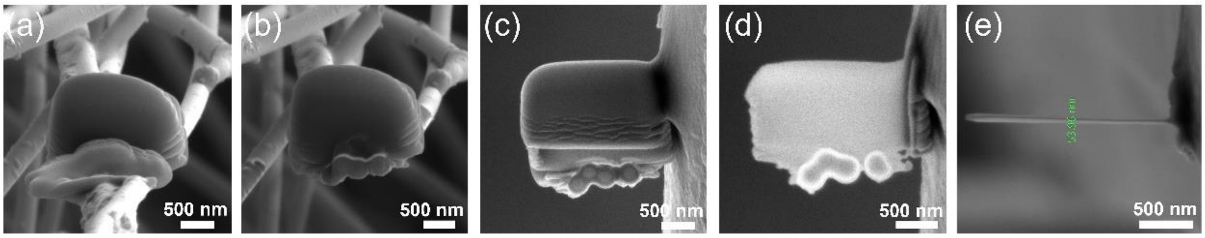

Fig. S2 The $SiC@SiO_2$ nanofibers cross-section preparation process. **a** Select target area, **b** and **c** sample excavation on the front and rear sides of the target location, **d** focused ion beam thinning of sample, and **e** the thinned sample thickness is only 53.69 nm

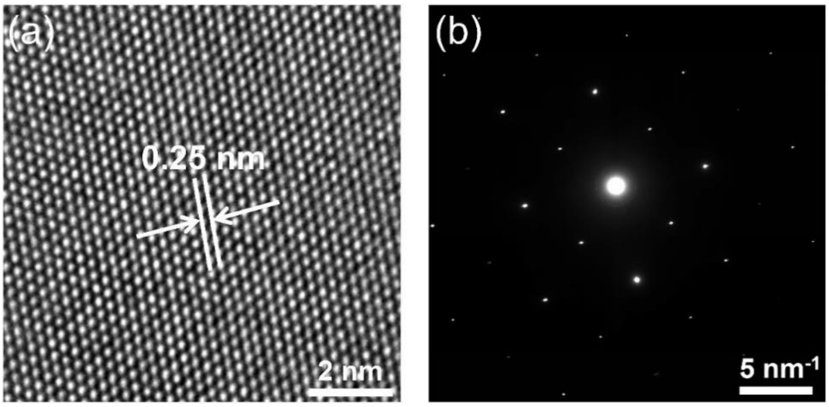

Fig. S3 High-resolution TEM (HR-TEM) of SiC core of the SiC@SiO_2 nanofibers and corresponding selected area electron diffraction (SAED)

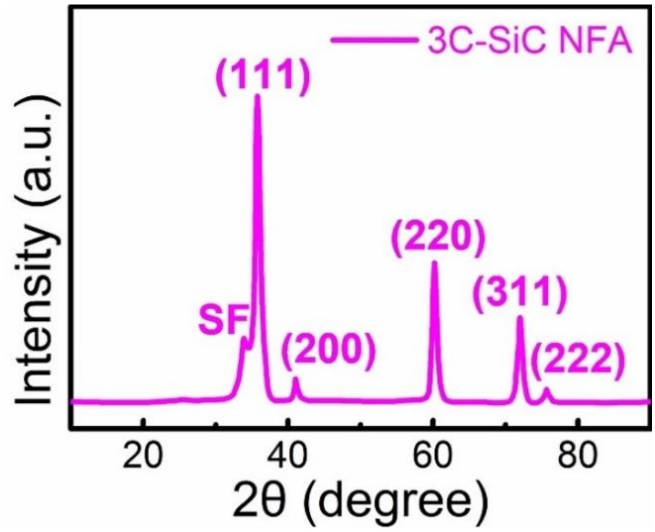

Fig. S4 XRD patterns for the SiC NFA

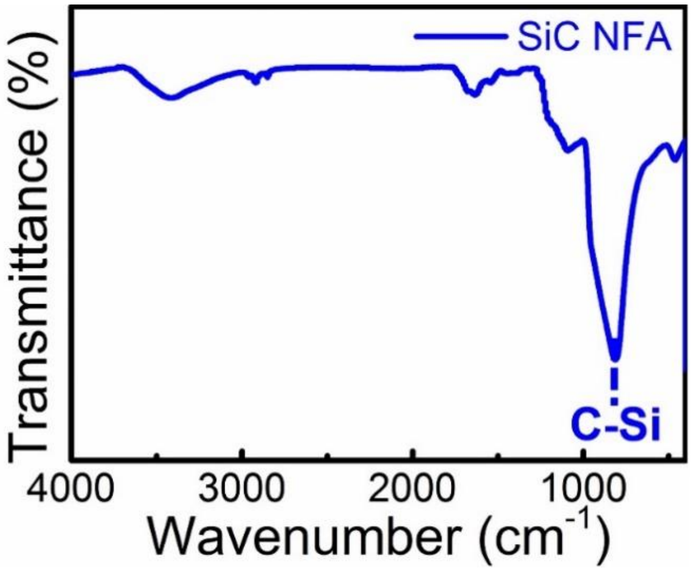

Fig. S5 FTIR spectra for the SiC NFA

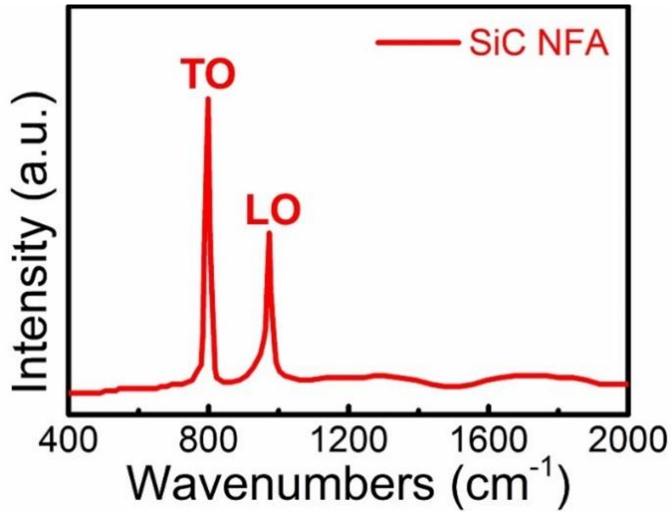

Fig. S6 Raman spectra for the SiC NFA

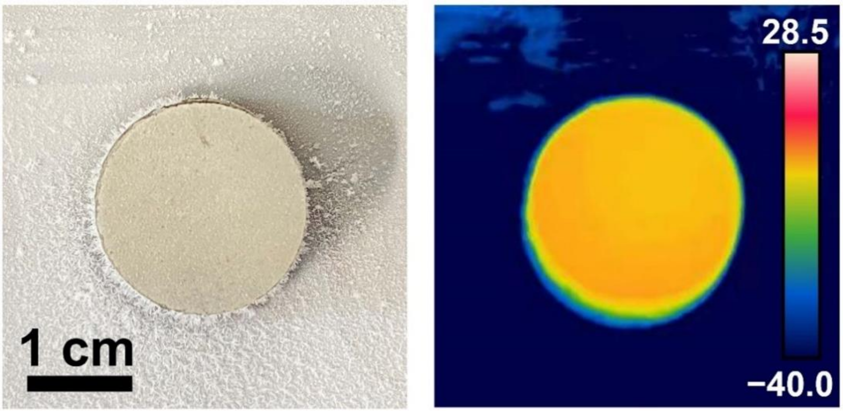

Fig. S7 The SiC@SiO_2 NFA on an iron plate above a jar filled with liquid nitrogen (~ −40 °C)

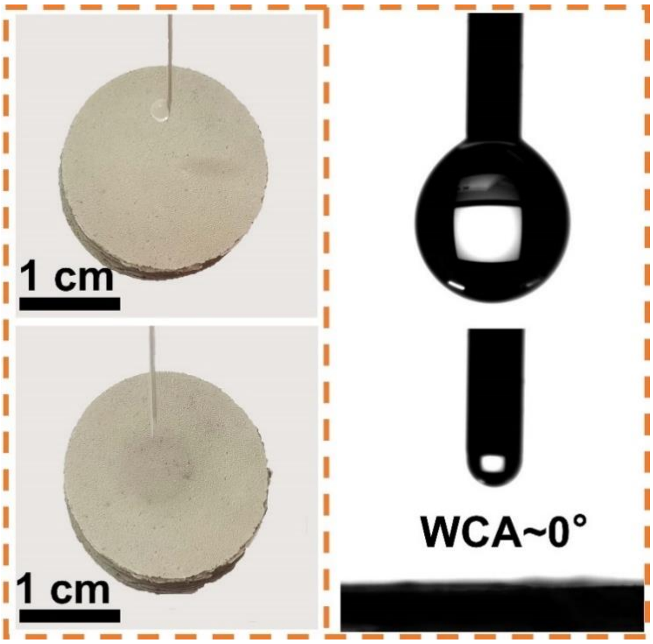

Fig. S8 The hydrophilicity of original SiC NFA with a water contact angle (WCA) of ~0°

The EMW-absorbing properties of dielectric loss materials are determined by their relative complex permittivities (ε_r). It is well known that the real part of the permittivity (ε') represents the storage capacity of electrical energy, while the imaginary part of the permittivity (ε'') indicates the ability to dissipate electrical energy [S1]. As shown in Fig. S9a, the value of real permittivity for the $SiC@SiO_2$ NFA gradually decreases from 14.47 to 7.75 in the frequency range of 2−12.46 GHz. The reason for this is that the rearrangement of the dipoles cannot match the increase in the frequency of the EMW, which leads to a weakening of the dielectric polarization effect. The specific manifestation is that the dielectric response decreases with increasing frequency [S2]. In addition, the ε' curve for the $SiC@SiO_2$ NFA shows some fluctuation in the frequency range of 12.46–18 GHz, which may be due to the dipole polarization caused by the orientation of some random dipoles in the material parallel to the EM field [S3]. Figure S9b shows that the value of ε'' gradually increases when the frequency increases from 2 to 11.6 GHz, which may be due to polarization relaxation. Furthermore, the value of ε'' gradually decreases with increasing frequency from 11.6 to 15.9 GHz, which may be due to the weakening of the interfacial polarization among the $SiC@SiO_2$ nanofibers in this frequency range. Interestingly, the curve for ε'' shows a broad upward peak in the frequency range of 15.9–18 GHz with a maximum value of 6.08, indicating an enhanced dipole polarization effect for the $SiC@SiO_2$ NFA. The dielectric tangent loss value ($\tan\delta_\varepsilon = \varepsilon''/\varepsilon'$) is generally used to indicate the energy dissipation capability. The variation trend for $\tan\delta_\varepsilon$ in the frequency range of 2–18 GHz is similar to that for ε'', as shown in Fig. S9c. The $\tan\delta_\varepsilon$ curve for the $SiC@SiO_2$ NFA exhibits two peaks at 12.07 and 16.28 GHz, suggesting that the aerogel has a superior dielectric loss performance in the high frequency range.

According to Debye theory [S4], the relative complex permittivity (ε_r) can be expressed as follows [S5]:

$$\varepsilon_r = \varepsilon' - j\varepsilon'' = \varepsilon_\infty + \frac{\varepsilon_s - \varepsilon_\infty}{1 + j2\pi f\tau} \qquad \text{(S1)}$$

where ε_s is the static permittivity, ε_∞ is the relative permittivity at infinite frequency, f is the frequency, and τ is the polarization relaxation time. Therefore, ε' and ε'' can be expressed as follows:

$$\varepsilon' = \varepsilon_\infty + \frac{\varepsilon_s - \varepsilon_\infty}{1 + \omega^2\tau^2} \qquad \text{(S2)}$$

$$\varepsilon'' = \frac{\varepsilon_s - \varepsilon_\infty}{1 + \omega^2\tau^2}\omega\tau + \frac{\sigma}{\omega\varepsilon_0} \qquad \text{(S3)}$$

$$tan\delta_\varepsilon = \frac{\varepsilon''}{\varepsilon'} = \frac{(\varepsilon_s - \varepsilon_\infty)2\pi f\tau}{\varepsilon_s + \varepsilon_\infty(2\pi f)^2\tau^2} \qquad \text{(S4)}$$

According to Eqs. (2) and (3), the relationship between ε' and ε'' can be expressed as follows:

$$\left(\varepsilon' - \frac{\varepsilon_s + \varepsilon_\infty}{2}\right)^2 + (\varepsilon'')^2 = \left(\frac{\varepsilon_s - \varepsilon_\infty}{2}\right)^2 \qquad \text{(S5)}$$

Based on Eq. (5), the Cole-Cole semicircle diagrams can be plotted, which relate the real and imaginary parts of the complex permittivity, and each semicircle represents a Debye relaxation process [S6, S7]. Figure S9d shows that there are at least three distinct semicircles, indicating the existence of multiple dielectric relaxation processes in the $SiC@SiO_2$ NFA.

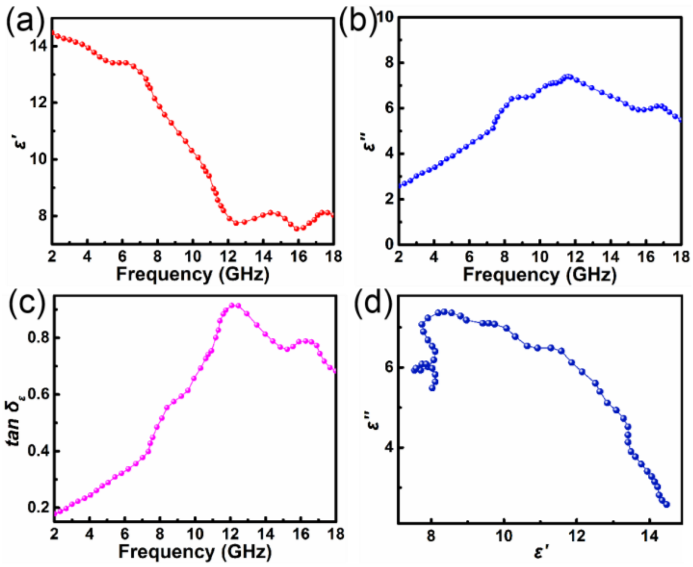

Fig. S9 Electromagnetic parameters for the SiC@SiO_2 NFA. (**a**) Real part of the permittivity, (**b**) imaginary part of the permittivity, (**c**) tangent loss, and (d) the Cole-Cole semicircle curves (ε″-ε′) for the SiC@SiO_2 NFA

Table S1 Multifunctional properties of the SiC@SiO_2 NFA

**Sample**	**Density (mg/cm^3)**	**Porosity (%)**	**BET specific surface areas (m^2 g^{-1})**	**Average pore size (nm)**	**RL_{min} (dB)**	**EAB_{max} (GHz)**
SiC@SiO_2 NFA	~11	~99.6	185.3	22	−50.36	8.6

Table S2 Mechanical performances of the SiC@SiO_2 NFA

**Cycles**	**Maximum stress (kPa)**				**Young's modulus (kPa)**			
	~25 °C	~700 °C	~ −40 °C	~ −196 °C	~25 °C	~700 °C	~ −40 °C	~ −196 °C
1	29.33	28.56	28.27	27.35	41.17	40.59	39.98	39.13
50	29.25	28.49	28.19	27.27	40.99	40.37	39.75	38.89
100	29.19	28.41	28.11	27.18	40.69	40.11	39.48	38.67
200	29.06	28.27	27.92	27.02	40.26	39.58	39.03	38.33
400	28.84	28.03	27.56	26.71	39.36	38.55	37.89	37.36
600	28.61	27.78	27.26	26.43	38.57	37.56	36.82	36.28
800	28.36	27.55	27.01	26.19	37.87	36.58	35.71	35.07
1000	28.12	27.31	26.8	25.95	37.08	35.5	34.75	34.06

Table S3 Thermal conductivities of the SiC@SiO_2 NFA

**Temperature (°C)**	20	200	400	600
**Thermal conductivity (W/(m·k))**	0.027	0.041	0.069	0.105

Supplementary References

[S1]F. Wu, K. Yang, Q. Li, T.R. Shah, M. Ahmad et al., Biomass-derived 3D magnetic porous carbon fibers with a helical/chiral structure toward superior microwave absorption. Carbon **173**, 918−931 (2021). https://doi.org/10.1016/j.carbon.2020.11.088

[S2]C.Y. Liang, Z.J. Wang, Eggplant-derived SiC aerogels with high-performance electromagnetic wave absorption and thermal insulation properties. Chem. Eng. J. **373**, 598–605 (2019). https://doi.org/10.1016/j.cej.2019.05.076

[S3]L. Yan, C. Hong, B. Sun, G. Zhao, Y. Cheng et al., In situ growth of core–sheath heterostructural SiC nanowire arrays on carbon fibers and enhanced electromagnetic wave absorption performance. ACS Appl. Mater. Interfaces **9**(7), 6320−6331 (2017). https://doi.org/10.1021/acsami.6b15795

[S4]Y.C. Du, W.W. Liu, R. Qiang, Y. Wang, X.J. Han et al., Shell thickness-dependant microwave absorption of core-shell Fe_3O_4@C composites. ACS Appl. Mater. Interfaces **6**(15), 12997–13006 (2014). https://doi.org/10.1021/am502910d

[S5]B. Wen, M.S. Cao, Z.L. Hou, W.L. Song, L. Zhang et al., Temperature dependent microwave attenuation behavior for carbon-nanotube/silica composites. Carbon **65**, 124−139 (2013). https://doi.org/10.1016/j.carbon.2013.07.110

[S6]X.L. Ye, Z.F. Chen, S.F. Ai, B. Hou, J.X. Zhang et al., Porous SiC/melamine-derived carbon foam frameworks with excellent electromagnetic wave absorbing capacity. J. Adv. Cream. **8**, 479−488 (2019). https://doi.org/10.1007/s40145-019-0328-2

[S7]Y. Cheng, J. Cao, Y. Li, Z. Li, H. Zhao et al., The outside-in approach to construct Fe_3O_4 nanocrystals/mesoporous carbon hollow spheres core–shell hybrids toward microwave absorption. ACS Sustain. Chem. Eng. **6**(1), 1427−1435 (2018). https://doi.org/10.1021/acssuschemeng.7b03846
